# Phyllodes tumors with and without fibroadenoma-like areas display distinct genomic features and may evolve through distinct pathways

**DOI:** 10.1038/s41523-017-0042-6

**Published:** 2017-10-12

**Authors:** Fresia Pareja, Felipe C. Geyer, Rahul Kumar, Pier Selenica, Salvatore Piscuoglio, Charlotte K. Y. Ng, Kathleen A. Burke, Marcia Edelweiss, Melissa P. Murray, Edi Brogi, Britta Weigelt, Jorge S. Reis-Filho

**Affiliations:** 1 Department of Pathology, Memorial Sloan Kettering Cancer Center, New York, NY USA; 2 Institute of Pathology, University Hospital Basel, Basel, Switzerland; 3 Department of Biomedicine, University of Basel, Basel, Switzerland

## Abstract

Breast fibroepithelial lesions (fibroadenomas and phyllodes tumors) are underpinned by recurrent *MED12* exon 2 mutations, which are more common in fibroadenomas and benign phyllodes tumors. *TERT* promoter hotspot mutations have been documented in phyllodes tumors, and found to be more frequent in borderline and malignant lesions. Several lines of evidence suggest that a subset of phyllodes tumors might arise from fibroadenomas. Here we sought to investigate the genetic differences between phyllodes tumors with fibroadenoma-like areas vs. those without. We retrieved data for 16 borderline/ malignant phyllodes tumors, including seven phyllodes tumors with fibroadenoma-like areas and nine phyllodes tumors without fibroadenoma-like areas, which had been previously subjected to targeted capture massively parallel sequencing. Whilst *MED12* exon 2 mutations were significantly more frequent in tumors with fibroadenoma-like areas (71 vs. 11%), an enrichment in genetic alterations targeting *bona fide* cancer genes was found in those without fibroadenoma-like areas, in particular in *EGFR* mutations and amplifications (78 vs. 14%). No significant difference in the frequency of *TERT* genetic alterations was observed (71% in cases with fibroadenoma-like areas vs 56% in those without fibroadenoma-like areas). Our data suggest that the development of phyllodes tumors might follow two different evolutionary pathways: a *MED12*-mutant pathway that involves the progression from a fibroadenoma to a malignant phyllodes tumor; and a *MED12*-wild-type pathway, where malignant phyllodes tumors arise de novo through the acquisition of genetic alterations targeting cancer genes. Additional studies are warranted to confirm our observations and define whether the outcome differs between both pathways.

## Genetics: Two paths to malignant fibroepithelial breast tumors

Malignant fibroepithelial breast tumors may arise by two distinct pathways: one from progression of benign tumors, and another where normal cells acquire mutations in cancer genes that result directly in the development of a more aggresive lesion. Phyllodes tumors (PTs) are rare breast tumors that start in the connective tissue; most are benign, but they are sometimes malignant. Jorge Reis-Filho from the Memorial Sloan Kettering Cancer Center in New York City, USA, and colleagues compared genetic differences between malignant PTs that resemble a benign tumor, known as fibroadenoma, and those that don’t. They found that PTs with fibroadenoma-like areas were more likely to harbor mutations in exon 2 of *MED12*, a gene often altered in benign breast fibroepithelial tumors, whereas PTs without fibroadenoma-like areas more commonly had mutations in *EGFR* and other known cancer genes. The findings suggest that some PTs arise from fibroadenomas, while others originate anew.

## Introduction

Fibroadenomas (FA) and phyllodes tumors (PT) are part of the spectrum of breast fibroepithelial lesions.^1^ Whilst FAs are benign tumors of frequent occurrence, which are usually managed conservatively, PTs are rare and may recur locally or even metastasize to distant sites.^2^ PTs are classified according to criteria recommended by the World Health Organization (WHO), which include tumor borders, stromal cellularity and atypia, mitotic activity and stromal overgrowth, into benign, borderline and malignant categories.^1^ Albeit not perfectly, the histologic grading of PTs is predictive of clinical behavior.^1^ Recurrence rates reported in the literature are of 10–17%, 14–25% and 23–30% for benign, borderline, and malignant PTs, respectively.^1^ Although metastases may occur in association to a PT of any grade, they are significantly more frequent following malignant PTs.^3–5^ PTs are managed with surgical excision and clear margins, with surgical margins status being an important predictor of clinical behavior.^3^

FAs and PTs share not only histologic similarities, but also genetic features. Highly recurrent and clonal somatic mutations targeting the exon 2 of *MED12* have been identified in both FAs and PTs,^6–11^ suggesting these constitute early founder events. Additional genes, such as *FLNA*, *SETD2* and *RARA*, are mutated in both entities but at a lower frequency in FAs.^11^ Importantly, among PTs, a stepwise decrease in the prevalence of *MED12* mutations from benign to borderline and to malignant PTs has been demonstrated.^10^ Moreover, whilst FAs have a less complex genomic landscape and lower mutational burden than PTs,^11^ somatic genetic alterations in *bona fide* cancer genes, such as *NF1*, *RB1*, *TP53*, *PIK3CA*, and *EGFR* appear to be restricted malignant and few borderline PTs.^10,11^ Finally, *TERT* promoter mutations and gene amplifications are absent or exceedingly rare in FAs, but recurrent in PTs, though significantly more frequent in borderline and malignant PTs than in benign PTs, suggesting that *TERT* genetic alterations might play a role in the progression from benign to malignant PTs.^8,10,12,13^

Malignant PTs are often histologically heterogeneous, displaying areas consistent with benign PTs or even FAs. Some studies have suggested that FAs may rarely progress to malignant PTs.^14–16^ Importantly, however, whilst grade progression within PTs is a well-described phenomenon,^17–19^ progression from FAs to PTs remains a matter of contention. Recent analyses of synchronous or metachronous ipsilateral fibroepithelial lesions revealed identical *MED12* mutations in pairs of FA and PT (benign and malignant), providing molecular evidence in support of the clonal relatedness between FAs and PTs from the same patients.^11,20^ In one patient with three FAs, one benign PT and one malignant PT in the same breast, we found that only the malignant PT, which was clonally related to one of the three FAs, harbored a *TERT* promoter mutation.^20^ These findings support the notion that FAs might constitute the substrate for the development of PTs, and that *TERT* genetic alterations might drive malignant progression in fibroepithelial lesions.^11,20^

Taken together, given that *MED12* mutations are significantly more common in benign fibroepithelial lesions than in malignant PTs, that FAs might constitute the substrate for the development of a subset of PTs and that *TERT* genetic alterations might drive malignant progression in fibroepithelial lesions, we hypothesized that the development of borderline and malignant PTs might follow two distinct evolutionary pathways, according to *MED12* status. In the *MED12*-mutant pathway, a linear progression from FA/ benign PT to borderline/ malignant PT would occur and be likely driven by the acquisition of *TERT* genetic alterations. By contrast, in the *MED12*-wild type pathway, borderline and malignant PTs are more likely to develop de novo, and driven by early genetic alterations in *TERT* and/or other cancer genes. To test this hypothesis, we investigated the differences in the repertoire of somatic genetic alterations of borderline and malignant PTs with FA-like areas, which might presumably have stemmed from a pre-existing FA, vs. that of PTs without FA-like areas, which might have arisen de novo.

## Results

**Fig. 1:**
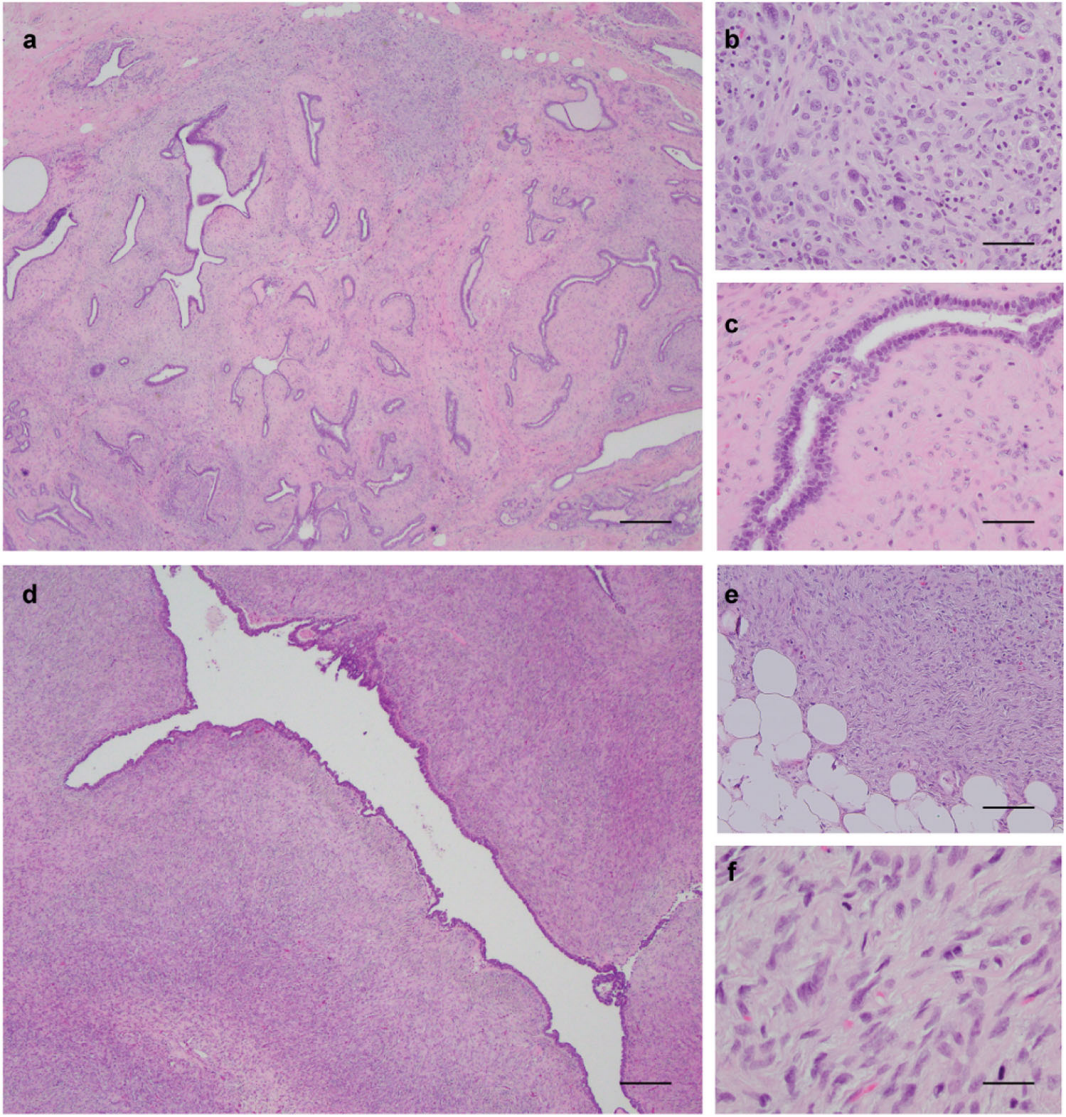
Histologic features of phyllodes tumors (PTs) with and without fibroadenoma (FA)-like areas included in this study. Representative micrographs of a malignant PT with FA-like areas **a**, displaying areas of marked stromal cellularity and cytologic atypia **b** intermingled with FA-like areas showing intracanalicular growth pattern, low stromal cellularity and no cytologic atypia **c**. Representative micrographs of a malignant PT without FA-like areas **d**, displaying infiltrative borders, prominent stromal cellularity **e**, marked cellular atypia and numerous mitoses **f**. Scale bars in **a** and **d**, 100 μm, **b**, **c**, and **f**, 20 μm, and **e**, 50 μm

Our study cohort consists of 16 PTs, previously described by Piscuoglio et al.,^10^ which were categorized by six pathologists with expertise in breast pathology (FP, FCG, ME, MM, EB, and JSR-F) as five borderline PTs and 11 malignant PTs according to WHO criteria.^1^ Only cases with all histologic slides available for re-review were included in this study. The median number of sections reviewed was 21 (range 8–42). In addition, all histological sections were re-reviewed by two pathologists (FP and FCG) to assess the presence of FA-like areas, defined as intracanalicular, pericanalicular or myxoid areas of low stromal cellularity, lacking cytologic atypia and mitotic activity. Discordant cases were reviewed on a multi-headed microscope together with a third pathologist (JSR-F) to reach a consensus. Based on the criteria above, seven PTs (five borderline and two malignant) were classified as having FA-like areas; nine PTs, all of them malignant, had no FA-like areas (Fig. 1). The number of sections reviewed per case was comparable between the two groups, (*p* > 0.05, Mann–Whitney *U* test).

We first compared the two groups (PTs with vs. without FA-like areas) in regards to their clinico-pathological features. The median age of the patients with PTs with FA-like areas was 43 years (range 28–55), younger but not significantly different from that of patients with PTs without FA-like areas (median age 58, range 25–76, *p* > 0.05, Mann–Whitney *U* test). Likewise, the median size of the PTs was similar between the group of PTs with FA-like areas, 9 cm (range 1.6–11), and the group of PTs without FA-like areas, 8.5 cm (range 1.6–23, *p* > 0.05, Mann–Whitney *U* test). With regard to the histopathologic features used for the classification of PTs (i.e., stromal cellularity, cytologic atypia, mitotic activity, stromal overgrowth, heterologous elements, and tumor borders), only mitotic activity differed significantly between the two groups, with PTs without FA-like areas having more mitoses than PTs with FA-like areas (*p* < 0.05, Fisher’s exact test; Table 1).

**Table 1:** Histologic features of phyllodes tumors according to the presence of fibroadenoma-like areas

Histologic parameter		PT with FA-like areas (*n*=7)	PT without FA-like areas (*n*=9)	*p* value
Tumor border	Well defined	4 (57%)	3 (33%)	0.592
	Permeative	3 (43%)	6 (67%)	
Stromal cellularity	Mild	1 (14%)	0 (0%)	0.212
	Moderate	4 (57%)	3 (33%)	
	Marked	2 (29%)	6 (67%)	
Stromal atypia	Mild	1 (14%)	1 (11%)	0.633
	Moderate	5 (71%)	4 (44%)	
	Marked	1 (14%)	4 (44%)	
Mitoses/10 HPF	0–10	6 (86%)	2 (22%)	0.04
	>10	1 (14%)	7 (78%)	
Stromal overgrowth	Absent	6 (86%)	5 (56%)	0.308
	Present	1 (14%)	4 (44%)	
Malignant heterologous elements	Absent	7 (100%)	7 (78%)	0.475
	Present	0 (0%)	2 (22%)	

*FA* fibroadenoma, *PT* phyllodes tumor

*p*-value according to Fisher’s exact test

The massively parallel sequencing data of all cases was retrieved the NCBI Sequence Read Archive under Accession No. SRP062618. Briefly, DNA samples from tumor and matching normal tissue had been subjected to high-depth targeted-capture massively parallel sequencing using the Memorial Sloan Kettering-Integrated Mutation Profiling of Actionable Cancer Targets (MSK-IMPACT) platform,^21^ which interrogates coding regions of up to 410 genes, as well as intronic regions and promoters of selected genes. MSK-IMPACT sequencing yielded a median depth of coverage of tumor samples of 556x (range 332x–754x; Supplementary Table 1). No significant difference in depth of coverage was observed between the two groups (*p* > 0.05, Mann–Whitney *U* test). Analysis of the MSK-IMPACT data revealed a similar number of non-synonymous somatic mutations in the PTs regardless of the presence of FA-like areas (medians of 4 (range 3–7) and 4 (range 2–8)) in PTs with FA-like areas and PTs without FA-like areas, respectively (*p* > 0.05, Mann–Whitney *U* test; Supplementary Table 2).

**Fig. 2:**
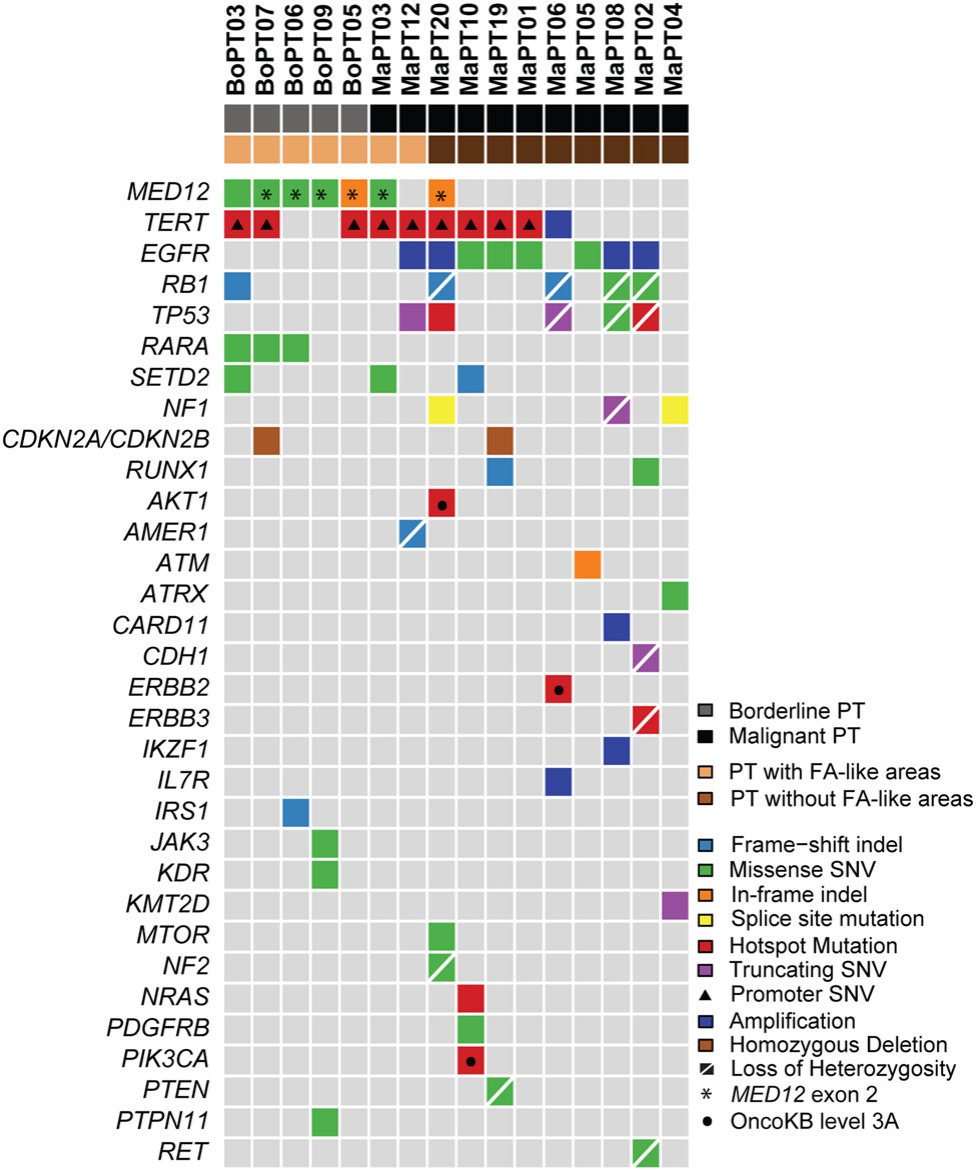
Repertoire of somatic genetic alterations identified in phyllodes tumors (PTs) with fibroadenoma (FA)-like areas and PTs without FA-like areas. Heatmap illustrating non-synonymous somatic mutations, gene amplifications and homozygous deletions in PTs with FA-like areas (*n* = 7) and PTs without FA-like areas (*n* = 9), identified by targeted capture massively parallel sequencing (MSK-IMPACT). Cases are shown in columns and genes are represented in rows. Only genetic alterations affecting the 227 genes present in both targeted capture panels used in this study are shown. The different genetic alterations are color-coded according to the legend. Loss of heterozygosity of a mutated gene is indicated by a diagonal bar. Promoter SNVs are shown with a triangle. *MED12* exon 2 mutations are indicated by an asterix; BOPT3 harbored a *MED12* mutation in exon 4. OncoKB level 3A (breast cancer) is indicated by a circle

Next we compared the difference in the frequency of mutations affecting single genes between the two groups. *MED12* exon 2 mutations were significantly more frequent in PTs with FA-like areas (5/7, 71%) than in PTs without FA-like areas (1/9, 11%; *p* < 0.05; Fisher’s exact test; Fig. 2 and Supplementary Table 2). *MED12* exon 2 mutations were predicted to be clonal by ABSOLUTE (i.e. present in virtually 100% of tumor cells analyzed in each sample) in all PTs with FA-like areas cases (Supplementary Fig. 1). In the sole *MED12*-mutant PT without FA-like areas, the *MED12* exon 2 mutation was subclonal, suggesting that it might not be the main genetic driver of this malignant PT at that evolutionary stage (Supplementary Fig. 1 and Supplementary Table 2). The frequency of *TERT* promoter mutations or amplifications was similar between the two groups, 5/7 (71%) in PTs with FA-like areas and 5/9 (56%) in PTs without FA-like areas (*p* > 0.05, Fisher’s exact test). Conversely, somatic genetic alterations affecting *EGFR*, including amplifications and likely pathogenic missense mutations, were significantly more frequent in PTs without FA-like areas (7/9, 78%) than in PTs with FA-like areas (1/7, 14%, p < 0.05, Fisher’s exact test; Fig. 2). The *EGFR* mutations targeted the ligand binding and tyrosine kinase domains, including the G63R, L62R, V774M, and E84V mutations. Furthermore, somatic genetic alterations in additional *bona fide* tumor suppressor genes, such as *RB1*, *TP53*, and *NF1*, often coupled with loss of heterozygosity of the wild-type allele, were more frequent in PTs without FA-like areas than in PTs with FA-like areas (Fig. 2), however these differences did not reach statistical significance. We cannot rule out that the lack of significant differences in the frequency of mutations affecting these genes is due to the small number of cases in our cohort.

**Fig. 3:**
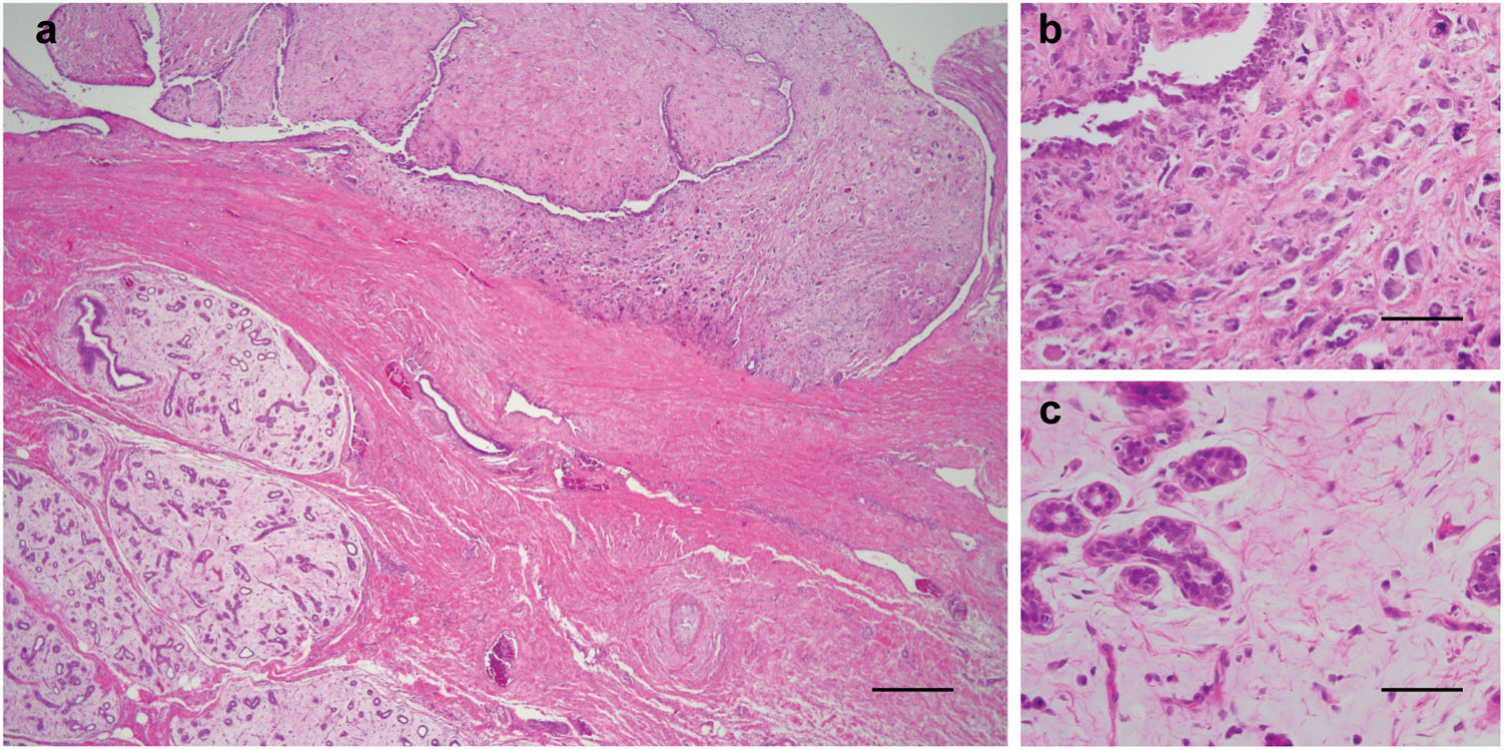
Histologic features of a phyllodes tumor (PT) with myxoid fibroadenoma (FA)-like areas. Representative micrographs of a malignant PT with myxoid FA-like areas **a**, displaying regions with marked stromal atypia and frequent mitoses **b** immediately adjacent to FA-like areas with myxoid hypocellular stroma **c**. Scale bar in a 100 μm, and b and c, 50 μm

The sole case of a PT with FA-like areas that was *MED12*-wild-type corresponded to a malignant PT associated to a myxoid FA (Fig. 3). We have previously observed that myxoid FAs, unlike conventional FAs, do not harbor *MED12* exon 2 mutations.^22^

Importantly, potentially targetable mutations, including the *AKT1* E17K, *ERBB2* V777L, *ERBB3* V104L, *NRAS* Q61K, and *PIK3CA* H1047R hotspot mutations, were restricted to PTs without FA-like areas (Fig. 2). Although no clinical evidence is currently available in terms of their actionability in PTs or sarcomas, clinical data support the potential targeting of these *AKT, ERBB2*, and *PIK3CA* hotspot mutations in breast cancer (OncoKB level of evidence 3A;^23^ Fig. 2 and Supplementary Table 3).

## Discussion

Even though PTs have been classically regarded as de novo lesions arising from specialized intra-lobular mammary stroma, their morphologic and genetic overlap with FAs, the frequent observation of FA-like areas in a subset of PTs, and the available molecular evidence demonstrating clonal relatedness between FAs and PTs in a subset of tumors, suggest that PTs and FAs might share a common origin.^1,11,2,20^ Conversely, however, our results and previous studies^10,11,13,8,24^ demonstrate that a substantial proportion of *bona fide* malignant PTs lack *MED12* exon 2 mutations, which are present in the large majority of FAs and benign PTs. These findings suggest that *MED12*-wild-type malignant PTs may constitute neoplasms distinct from PTs associated with FAs or that these lesions evolve through distinct evolutionary paths. Indeed, here we demonstrate that borderline and malignant PTs with FA-like areas display a significantly different genomic landscape than PTs without FA-like areas. Whilst PTs with FA-like areas harbor highly recurrent *MED12* exon 2 mutations and less common genetic alterations in *bona fide* cancer genes, most PTs without FA-like areas are *MED12*-wild-type and display more frequent genetic alterations targeting cancer genes, in particular *EGFR* (Fig. 2).

**Fig. 4:**
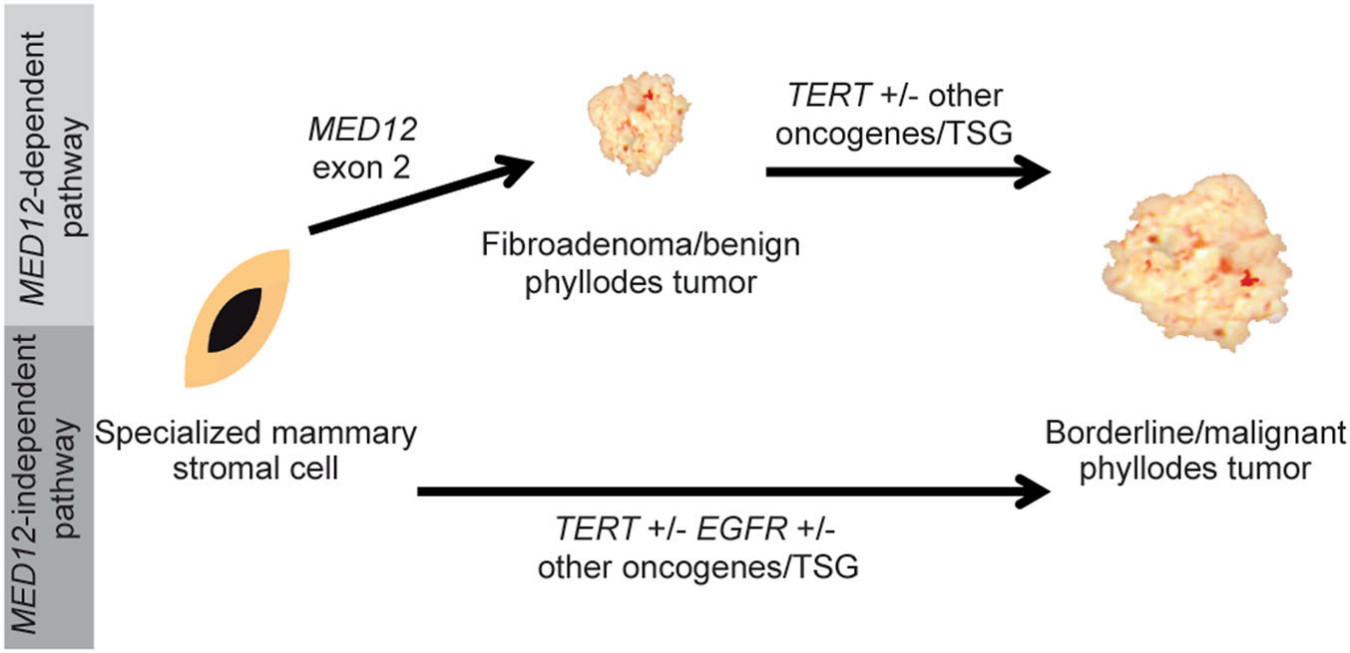
Proposed model of the evolutionary origin of borderline and malignant phyllodes tumors. Phyllodes tumors might follow two different evolutionary pathways. (i) In the *MED12*-mutant pathway, *MED12* exon 2 mutations are posited to lead to the development of a benign fibroepithelial lesion, which upon the occurrence of additional genetic alterations affecting *TERT* and/ or other cancer genes may progress to a borderline or malignant phyllodes tumor. (ii) In the *MED12*-independent pathway, borderline or malignant phyllodes tumors might arise *de novo*, through the acquisition of genetic alterations targeting cancer genes, such as *TERT* and/ or *EGFR*. TSG tumor suppresor genes

Our findings support the hypothesis that borderline and malignant PTs may develop following two different evolutionary pathways. In the setting of the *MED12*-mutant pathway, borderline and malignant PTs might stem from a pre-existing FA and progress to a malignant phenotype following the acquisition of additional genetic alterations in cancer genes, including *TERT* promoter mutations and *TERT* amplification. In the context of the *MED12-*independent pathway, malignant PTs might arise *de novo* following the early acquisition of genetic alterations affecting cancer genes, including not only *TERT* genetic alterations, but, most strikingly, activation of oncogenes, such as *EGFR*, and/ or loss of function of tumor suppressor genes, such as *RB1* and *TP53* (Fig. 4). Consistent with our genetic findings, clinical studies have suggested the existence of two subsets of PTs with different prognosis depending on a previous history of ipsilateral FA or the finding of *MED12* somatic mutations.^25,26^ Abe et al.^25^ reported that the overall survival of patients with malignant PTs and a history of ipsilateral FA was significantly longer than the one of patients without a history of FA. Ng et al.^26^ observed that PTs harboring *MED12* mutations have a lower recurrence rate than those without *MED12* mutations. Based on these findings, it could be posited that PTs with a previous history of FA or with FA-like areas may be driven by *MED12* and associated with a more favorable clinical outcome, whereas PTs without a history of FA or FA-like areas develop in a *MED12*-independent manner and may have a worse outcome.

It is noteworthy that the single *MED12*-mutant PT without FA-like areas harbored a subclonal *MED12* mutation, indicating intra-tumor heterogeneity, whereas in all the other *MED12*-mutant cases, the *MED12* mutation was clonal and FA-like areas were present (Fig. 2, Supplementary Fig. 1 and Supplementary Table 2). This case harbored an *EGFR* amplification, as well as clonal mutations targeting cancer genes, such as the *TP53* R249S and *AKT1* E17K hotspot mutations. Potential explanations include that a *MED12*-mutant FA and a *MED12*-wild type malignant PT originated independently, and that growth of the latter obliterated the FA areas. Alternatively, the PT might have originated from a pre-existing FA harboring a *MED12* mutation, that was overgrown by the malignant component driven by *EGFR* and *AKT1* activation and loss of function of p53, in which *MED12* mutation was not required any longer for tumor growth.

Importantly, we have shown that PTs with and without FA-like areas differ in terms of the somatic genetic alterations that are potentially targetable, as hotspot mutations in *AKT1* (E17K), *ERBB2* (V777L), *ERBB3* (V104L), *NRAS* (Q61K), and *PIK3CA* (H1047R) were found to be exclusive to PTs without FA-like areas (Fig. 2). Even though the therapeutic relevance of these findings in PTs is currently unknown, clinical evidence supports the role of the majority of these mutations as predictors of response to specific therapeutic agents in other malignancies.^27–31^ In fact, AZD5363, an AKT inhibitor has been shown to have therapeutic activity in breast and ovarian cancer harboring the *AKT1* E17K mutation,^27^ and neratinib, an irreversible dual ERBB2/EGFR tyrosine kinase inhibitor, has been found to be active in breast cancers with activating *ERBB2* mutations.^28^

Although there is currently no compelling clinical evidence supporting the role of *EGFR* amplifications and the missense mutations identified in this study (i.e., L62R, G63R, E84V, and V774M) as biomarkers predictive of therapeutic response, these genetic alterations could be potentially targetable. *EGFR* amplifications and the V774M tyrosine kinase mutation are known to be oncogenic,^32^ and the L62R, G63R, and E84V mutations have been recently identified as new functional variants,^33^ and target the ligand domain of EGFR, which plays a crucial role in its dimerization and activation, and is currently the target of anti-EGFR monoclonal antibodies such as cetuximab and panitumumab.^34^

Our study has important limitations. First, our series did not include borderline PTs without FA-like areas. However, our findings may be a reflection of the fact that PTs that stem from pre-existing FAs might be of lower grade than those developing de novo. Second, the assessment of the differences in clinical behavior between PTs with FA-like areas and those without FA-like areas was not possible due to the small size of our cohort, low recurrence rate of the lesions and short follow-up time. Third, sampling of specimens included in the study was performed for clinical purposes and not tailored for the assessment of the presence of FA-like areas. Nonetheless, we only included cases for which all histologic sections were available for re-review. Fourth, the cases in our study were analyzed using the MSK-IMPACT platform, which interrogates a limited number of genes; therefore it is possible that genes not included in this platform (e.g., *FLNA*) might be drivers not recognized in our study. Finally, we cannot exclude the possibility that at least a subset of PTs without FA-like areas could have originated from a pre-existing FA, in which the malignant component outgrew and obliterated a pre-existing FA.

Despite the limitations of our study, our data support the notion that the development of PTs may follow two different evolutionary pathways; a concept that appears to be supported by histologic features and *MED12* mutation status. PTs that may have stemmed from a pre-existing benign fibroepithelial lesion, such as a FA, retain FA-like areas and *MED12* exon 2 mutations, which were presumably present in the benign fibroepithelial lesion that they stemmed from. A different subset of borderline and malignant PTs might have arisen de novo through the acquisition of somatic genetic alterations in cancer genes such as *EGFR*. Further studies are required to determine whether the different components of PTs harboring FA-like areas are clonally related, as well as large longitudinal studies to determine the genetic alterations responsible for the progression of benign fibroepithelial lesions to borderline and malignant PTs.

## Methods

### Cases

Here we performed a reanalysis of the massively parallel sequencing data of five borderline PTs and 11 malignant PTs described in our previous study by Piscuoglio et al.^10^ The current study focuses on borderline and malignant PTs with and without FA-like areas. The cases were classified as PTs with FA-like areas or PTs without FA-like areas following the re-review of all cases by six pathologists with expertise in breast pathology. We did not include the benign PTs from the study by Piscuoglio et al.^10^ In addition, one borderline and two malignant PTs from Piscuoglio et al.^10^ were excluded from this re-analysis, given that only a subset of the histologic slides were available for re-review, and, therefore, the presence of FA-like areas could not be ruled out.

### Targeted-capture massively parallel sequencing

A detailed description of tissue microdissection, DNA extraction and sequencing is provided in the original publication.^10^ The massively parallel sequencing data of the relevant cases were retrieved from the NCBI Sequence Read Archive under Accession No. SRP062618. Tumor and normal DNA from all borderline PTs and nine malignant PTs had been analyzed at the Memorial Sloan Kettering Cancer Center Integrated Genomics Operation (MSKCC IGO) using the MSK-IMPACT sequencing assay, which interrogates all coding regions and selected intronic regions and promoters of up to 410 key cancer genes. Detection of somatic mutations was performed as detailed in Piscuoglio et al.^10,35–40^ Allele-specific copy number alterations were identified using FACETS.^41^ Targeted massively parallel sequencing of cases MaPT19 and MaPT20 had been performed using MSK-IMPACT v.3, which interrogates all coding exomes and selected non-coding regions of 275 genes. Detection of somatic mutations and copy number alterations for these cases had been performed as described in Cheng et al.^21^ The 227 genes common to MSK-IMPACT (410 genes) and MSK-IMPACT v.3 were considered in the re-analysis described here and are listed in the Supplementary Table 4. The likelihood of a mutation to be regarded as pathogenic had been assessed by the integration of the combination of mutation function predictors^42–45^ and the presence of the mutated gene in the cancer gene lists by Kandoth et al.^46^ the Cancer Gene Census^47^ or Lawrence et al.^48^ as detailed in Piscuoglio et al.^10^ The cancer cell fraction (CCF) of each mutation was defined using ABSOLUTE (v1.0.6)^49,50^ as inferred in Piscuoglio et al.^10^ Mutations were considered clonal if their probability of being clonal was >50%^50^ or if the lower bound of the 95% confidence interval of its CCF was >90%, otherwise the mutations were considered subclonal.

### Statistical analysis

Comparisons of depth of coverage and mutation rates were performed using the Mann–Whitney *U* test. Differences in the frequency of genetic alterations affecting single genes were assessed using Fisher’s exact test. Two-tailed *p*-values < 0.05 were considered statistically significant.

### Data availability

Sequencing data is available at the National Center for Biotechnology Information Sequence Read Archive under the accession SRP062618. All relevant data are available from the authors.

## Electronic supplementary material

Supplementary Legends

Supplementary Figure 1

Supplementary Table 1

Supplementary Table 2

Supplementary Table 3

Supplementary Table 4
