# Supplementary material for: Phyllodes tumors with and without fibroadenoma-like areas display distinct genomic features and may evolve through distinct pathways
Source: NPJ Breast Cancer. 2017 Oct 12;3:40. doi: 10.1038/s41523-017-0042-6 (PMC5638820; doi:10.1038/s41523-017-0042-6)
Supplement: Supplementary file 1 — Supplementary Legends [file 41523_2017_42_MOESM1_ESM.pdf]

Phyllodes tumors with and without fibroadenoma-like areas display distinct genomic features and may evolve through distinct pathways

Supplementary Legends

Supplementary Figure 1. Repertoire of somatic genetic alterations identified in phyllodes tumors (PTs) with fibroadenoma (FA)-like areas and PTs without FA-like areas.
Heatmap illustrating the cancer cell fractions of non-synonymous somatic mutations in PTs with FA-like areas (n=7) and PTs without FA-like areas (n=9), identified by targeted capture massively parallel sequencing (MSK-IMPACT). Cases are shown in columns and genes are represented in rows. Only genetic alterations affecting the 227 genes present in both targeted capture panels used in this study are shown. Cancer cell fractions are color coded according to the legend. Loss of heterozygosity of a mutated gene is indicated by a diagonal bar.

Supplementary Table 1: Sequencing statistics.

Supplementary Table 2: Somatic mutations identified in the cases subjected to targeted capture massively parallel sequencing.

Supplementary Table 3: OncoKB clinical actionability level of evidence.

Supplementary Table 4: List of genes included in the MSK-IMPACT panels employed in this study.
