# Supplementary figures and images for: Phyllodes tumors with and without fibroadenoma-like areas display distinct genomic features and may evolve through distinct pathways
Source: NPJ Breast Cancer. 2017 Oct 12;3:40. doi: 10.1038/s41523-017-0042-6 (PMC5638820; doi:10.1038/s41523-017-0042-6)

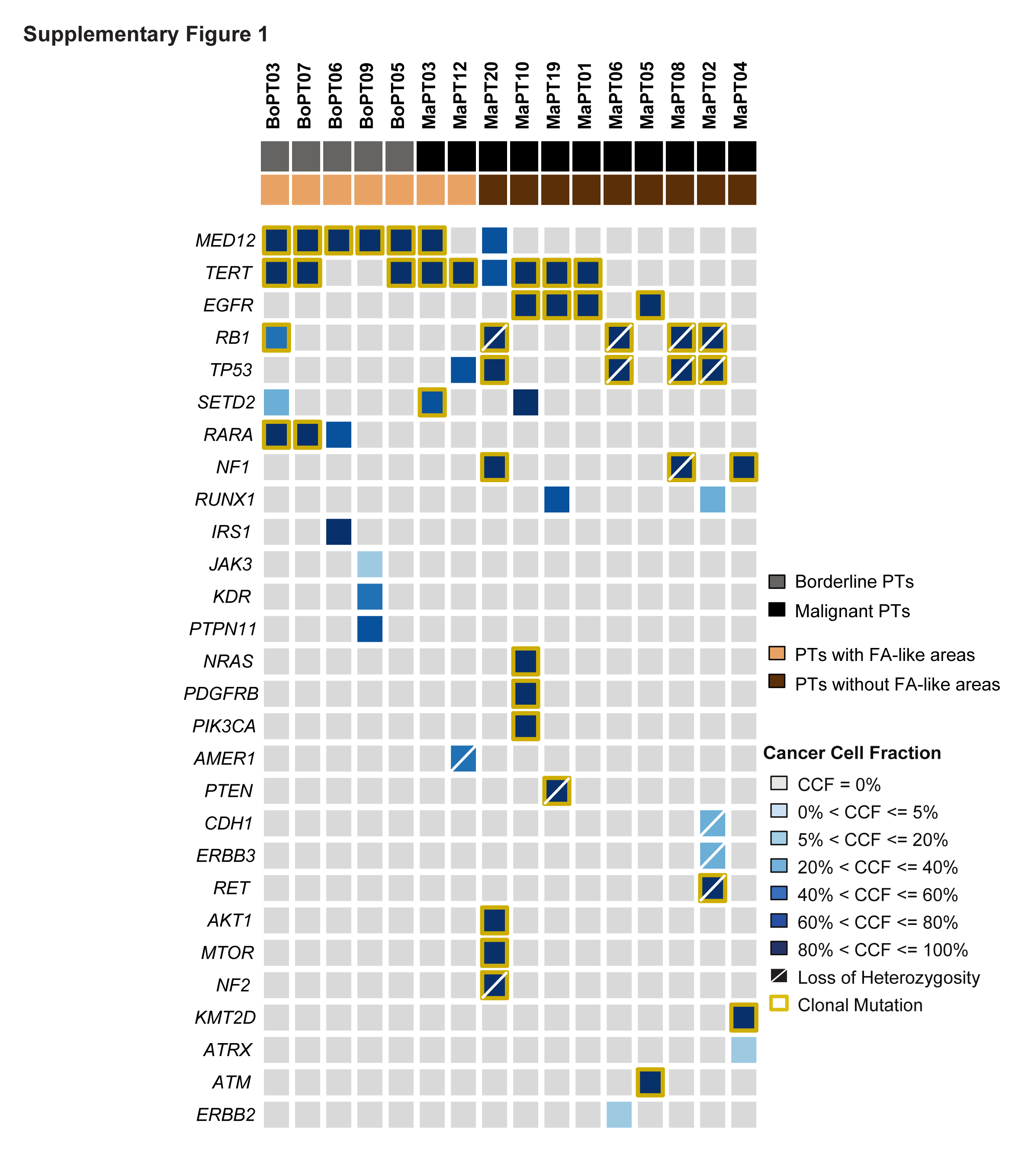

Supplement: Supplementary file 2 — Supplementary Figure 1 [file 41523_2017_42_MOESM2_ESM.tif]
