# Supplementary material for: Phyllodes tumors with and without fibroadenoma-like areas display distinct genomic features and may evolve through distinct pathways
Source: NPJ Breast Cancer. 2017 Oct 12;3:40. doi: 10.1038/s41523-017-0042-6 (PMC5638820; doi:10.1038/s41523-017-0042-6)
Supplement: Supplementary file 3 — Supplementary Table 1 [file 41523_2017_42_MOESM3_ESM.pdf]

Supplementary Table 1: Sequencing Statistics

Sample ID	Total Reads	Mean Target Coverage	% Target Bases 2X	% Target Bases 10X	% Target Bases 20X	% Target Bases 30X	% Target Bases 40X	% Target Bases 50X	% Target Bases 100X
BoPT3N	14,007,535	339.4	99.24%	98.96%	98.68%	98.42%	98.09%	97.71%	93.56%
BoPT3T	26,471,819	524.7	99.32%	99.07%	98.91%	98.77%	98.63%	98.49%	97.52%
BoPT5N	15,269,459	355.4	99.32%	99.06%	98.82%	98.61%	98.36%	98.04%	94.75%
BoPT5T	31,765,776	697.7	99.41%	99.17%	99.06%	98.96%	98.85%	98.75%	98.13%
BoPT6N	9,815,558	194.6	99.16%	98.70%	98.30%	97.84%	97.20%	96.22%	86.84%
BoPT6T	29,790,995	654.7	99.33%	99.14%	99.01%	98.88%	98.75%	98.63%	97.94%
BoPT7N	14,879,701	302.1	99.27%	98.95%	98.70%	98.46%	98.14%	97.77%	94.20%
BoPT7T	33,916,141	560.4	99.32%	99.07%	98.95%	98.81%	98.67%	98.56%	97.93%
BoPT9N	13,749,787	262.3	99.21%	98.79%	98.47%	98.13%	97.68%	97.08%	91.08%
BoPT9T	25,093,083	448.7	99.27%	98.99%	98.78%	98.59%	98.42%	98.26%	96.76%
MaPT2N	20,850,075	238.4	99.19%	98.63%	98.30%	98.05%	97.74%	97.29%	91.42%
MaPT2T	35,555,141	605.7	99.28%	99.02%	98.83%	98.67%	98.54%	98.42%	97.70%
MaPT3N	17,456,475	397.7	99.25%	99.00%	98.77%	98.57%	98.38%	98.17%	96.13%
MaPT3T	34,085,474	754.3	99.36%	99.13%	99.02%	98.92%	98.80%	98.69%	98.12%
MaPT1N	12,108,021	283.8	99.17%	98.79%	98.49%	98.16%	97.77%	97.22%	92.06%
MaPT1T	26,336,175	610.4	99.27%	99.06%	98.92%	98.77%	98.64%	98.50%	97.64%
MaPT4N	11,477,281	206.7	99.12%	98.66%	98.21%	97.68%	96.89%	95.82%	86.54%
MaPT4T	27,698,519	499.2	99.27%	99.01%	98.80%	98.62%	98.46%	98.29%	97.07%
MaPT5N	16,251,731	386.3	99.30%	99.03%	98.81%	98.61%	98.37%	98.08%	95.03%
MaPT5T	22,908,492	466.4	99.29%	99.08%	98.88%	98.71%	98.54%	98.36%	97.01%
MaPT6N	12,370,355	293.8	99.21%	98.88%	98.60%	98.31%	97.95%	97.41%	92.39%
MaPT6T	32,225,718	722.5	99.31%	99.10%	98.98%	98.84%	98.72%	98.61%	97.92%
MaPT8N	7,642,797	212	99.15%	98.74%	98.26%	97.49%	96.32%	94.79%	83.42%
MaPT8T	30,141,836	552.2	99.19%	98.82%	98.56%	98.41%	98.26%	98.12%	96.91%
MaPT10N	10,287,223	297.9	99.22%	98.89%	98.55%	98.17%	97.60%	96.88%	90.45%
MaPT10T	16,810,912	332	99.18%	98.75%	98.47%	98.26%	98.05%	97.79%	95.00%
MaPT12N	15,020,426	394.9	99.20%	98.96%	98.72%	98.49%	98.20%	97.75%	93.82%
MaPT12T	31,409,944	536.2	99.26%	99.01%	98.80%	98.64%	98.50%	98.36%	97.57%
MaPT19	33,619,544	451.2	99.24%	98.82%	98.48%	98.19%	97.95%	97.76%	96.62%
MaPT20	25,760,210	615.8	99.16%	98.84%	98.49%	98.23%	98.01%	97.79%	96.59%
