# Supplementary material for: Phyllodes tumors with and without fibroadenoma-like areas display distinct genomic features and may evolve through distinct pathways
Source: NPJ Breast Cancer. 2017 Oct 12;3:40. doi: 10.1038/s41523-017-0042-6 (PMC5638820; doi:10.1038/s41523-017-0042-6)
Supplement: Supplementary file 4 — Supplementary Table 2 [file 41523_2017_42_MOESM4_ESM.pdf]

Supplementary Table 2: Somatic mutations identified in the cases subjected to targeted capture massively parallel sequencing

Sample ID	Gene	Amino Acid Change	Effect	MAF	Depth	LOH Status	Cancer Cell Fraction (CCF) (ABSOLUTE)	Probability of mutation being clonal	Lower bound of 95% confidence interval	Clonal/ Subclonal mutation	Normal Depth	Chromosome	Position	Reference Allele	Alternate Allele	Mutation Taster	CHASM (Breast)	FATHMM	PROVEAN (in-frame indels only)	Kandoth et al 127 significantly mutated genes	Lawrence et al Cancer5000-S	Cancer Gene Census	Pathogenicity	Hotspot
BoPT03	*TERT*	c.-124C>T	upstream_gene_variant	38.40%	185		1	0.9618	0.9262	Clonal	110	5	1295228	G	A	.	.	.					Likely pathogenic	TRUE
BoPT03	*MED12*	p.Glu172Lys	missense_variant	27.90%	489		1	0.5058	0.9214	Clonal	370	X	70339981	G	A	D	Passenger	PASSENGER/OTHER			TRUE	TRUE	Non-pathogenic	FALSE
BoPT03	*RARA*	p.Cys235Trp	missense_variant	30.50%	494		1	0.0047	0.941	Clonal	346	17	38508657	C	G	D	Passenger	PASSENGER/OTHER				TRUE	Non-pathogenic	FALSE
BoPT03	*RB1*	p.Gly310fs	frameshift_variant	12.56%	286		0.58	0.001	0.4194		202	13	48939095	TG	T	A	.			TRUE	TRUE	TRUE	Likely pathogenic	FALSE
BoPT03	*RB1*	p.Ile848Arg	missense_variant	7.70%	182		0.35	0	0.209		139	13	49050859	T	G	D	Passenger	CANCER		TRUE	TRUE	TRUE	Likely pathogenic	FALSE
BoPT03	*RB1*	p.Pro250fs	frameshift_variant	20.41%	495		0.94	0.7658	0.7717	Clonal	337	13	48936979	ACC	A	A	.			TRUE	TRUE	TRUE	Likely pathogenic	FALSE
BoPT03	*SETD2*	p.Arg1625Cys	missense_variant	5.70%	319		0.26	0	0.1609		208	3	47144880	G	A	D	Driver	PASSENGER/OTHER		TRUE	TRUE	TRUE	Likely pathogenic	FALSE
BoPT05	*TERT*	c.-124C>T	upstream_gene_variant	16.50%	297		1	0.9383	0.8906	Clonal	110	5	1295228	G	A	.	.	.					Likely pathogenic	TRUE
BoPT05	*DOT1L*	p.Gly1471Glu	missense_variant	5.10%	313		0.59	0.1061	0.365		170	19	2226932	G	A	N	Passenger	PASSENGER/OTHER					Non-pathogenic	FALSE
BoPT05	*MED12*	p.Leu39_Asn47del	inframe_deletion	7.61%	914		0.89	0.6574	0.6997	Clonal	566	X	70339236	CCTTGAATGTAAAACAAGGTTTCAATAA	C	.	.		Deleterious		TRUE	TRUE	Likely pathogenic	FALSE
BoPT06	*ASXL1*	p.Glu1423Glu	synonymous_variant	3.20%	381		0.09	0	0.0436		108	20	31024784	G	A	.	.	.		TRUE	TRUE	TRUE	Non-pathogenic	FALSE
BoPT06	*EIF4A2*	p.Met38Ile	missense_variant	8.60%	835		0.23	0	0.182		280	3	186502391	G	A	D	Passenger	PASSENGER/OTHER		TRUE		TRUE	Non-pathogenic	FALSE
BoPT06	*IRS1*	p.Ala874fs	frameshift_variant	32.86%	514		0.89	0.4058	0.78		148	2	227660834	GC	G	A	.						Non-pathogenic	FALSE
BoPT06	*MED12*	p.Gly44Ala	missense_variant	38.20%	703		1	0.9348	0.9113	Clonal	228	X	70339254	G	C	D	Passenger	PASSENGER/OTHER			TRUE	TRUE	Non-pathogenic	FALSE
BoPT06	*RARA*	p.Leu398Val	missense_variant	29.30%	352		0.79	0.0353	0.6661		183	17	38512281	C	G	D	Passenger	PASSENGER/OTHER				TRUE	Non-pathogenic	FALSE
BoPT06	*TRAF7*	p.Asp357Glu	missense_variant	14.80%	176	LOH	0.25	0	0.1713		125	16	2223540	C	G	D	Passenger	PASSENGER/OTHER				TRUE	Non-pathogenic	FALSE
BoPT07	*TERT*	c.-124C>T	upstream_gene_variant	26.20%	261		1	0.9589	0.9087	Clonal	129	5	1295228	G	A	.	.	.					Likely pathogenic	TRUE
BoPT07	*MED12*	p.Gly44Val	missense_variant	36.10%	579		1	0.9705	0.9718	Clonal	360	X	70339254	G	T	D	Passenger	PASSENGER/OTHER			TRUE	TRUE	Non-pathogenic	FALSE
BoPT07	*RARA*	p.Ser287Leu	missense_variant	31.30%	600		1	0.9667	0.9647	Clonal	304	17	38510606	C	T	D	Passenger	PASSENGER/OTHER				TRUE	Non-pathogenic	FALSE
BoPT09	*JAK3*	p.Leu635His	missense_variant	3.30%	427		0.2	0	0.1182		139	19	17946743	A	T	D	Driver	CANCER				TRUE	Likely pathogenic	FALSE
BoPT09	*KDR*	p.Ala775Val	missense_variant	8.70%	241		0.46	0.0001	0.2985		146	4	55964913	G	A	D	Passenger	CANCER				TRUE	Likely pathogenic	FALSE
BoPT09	*MED12*	p.Gly44Val	missense_variant	19.00%	426		1	0.826	0.7879	Clonal	304	X	70339254	G	T	D	Passenger	PASSENGER/OTHER			TRUE	TRUE	Non-pathogenic	FALSE
BoPT09	*PTPN11*	p.Arg527His	missense_variant	10.10%	150		0.62	0.1395	0.3801		91	12	112926960	G	A	D	Passenger	PASSENGER/OTHER		TRUE	TRUE	TRUE	Non-pathogenic	FALSE
MaPT01	*TERT*	c.-124C>T	upstream_gene_variant	40.60%	217		1	0.9203	0.8533	Clonal	119	5	1295228	G	A	.	.	.					Likely pathogenic	TRUE
MaPT01	*EGFR*	p.Gly63Arg	missense_variant	38.90%	409		1	0.9299	0.879	Clonal	284	7	55210077	G	A	D	Driver	CANCER		TRUE	TRUE	TRUE	Likely pathogenic	FALSE
MaPT02	*ERBB3*	p.Val104Leu	missense_variant	5.70%	494	LOH	0.21	0	0.1434		233	12	56478854	G	T	D	Passenger	PASSENGER/OTHER			TRUE		Non-pathogenic	TRUE
MaPT02	*TP53*	p.Ser241Tyr	missense_variant	29.90%	374	LOH	1	0.9422	0.8793	Clonal	172	17	7577559	G	T	D	Driver	CANCER		TRUE	TRUE	TRUE	Likely pathogenic	TRUE
MaPT02	*CDH1*	p.Glu745*	stop_gained	8.00%	314	LOH	0.3	0	0.2005		132	16	68862145	G	T	A	.	.		TRUE	TRUE	TRUE	Likely pathogenic	FALSE
MaPT02	*FAT1*	p.Ser377Ser	synonymous_variant	4.50%	382		0.21	0	0.1287		133	4	187629851	A	G	.	.	.			TRUE		Non-pathogenic	FALSE
MaPT02	*RB1*	p.Cys706Phe	missense_variant	23.00%	282	LOH	0.87	0.6234	0.6884	Clonal	144	13	49037877	G	T	D	Driver	CANCER		TRUE	TRUE	TRUE	Likely pathogenic	FALSE
MaPT02	*RET*	p.Ser406Gly	missense_variant	34.10%	337	LOH	1	0.6382	0.9149	Clonal	159	10	43604631	A	G	N	Passenger	CANCER				TRUE	Non-pathogenic	FALSE
MaPT02	*RUNX1*	p.Asp93Tyr	missense_variant	5.00%	523		0.24	0	0.1578		344	21	36259214	C	A	D	Passenger	CANCER		TRUE	TRUE	TRUE	Likely pathogenic	FALSE
MaPT03	*TERT*	c.-124C>T	upstream_gene_variant	27.90%	181		1	0.9388	0.8784	Clonal	158	5	1295228	G	A	.	.	.					Likely pathogenic	TRUE
MaPT03	*MED12*	p.Gly44Asp	missense_variant	36.10%	714		1	0.7922	0.9779	Clonal	482	X	70339254	G	A	D	Passenger	PASSENGER/OTHER			TRUE	TRUE	Non-pathogenic	FALSE
MaPT03	*SETD2*	p.Asp1616His	missense_variant	28.00%	378		1	0.9263	0.9375	Clonal	183	3	47144907	C	G	D	Passenger	PASSENGER/OTHER		TRUE	TRUE	TRUE	Non-pathogenic	FALSE
MaPT03	*SETD2*	p.Ser1777Phe	missense_variant	11.40%	406		0.71	0.0746	0.5335		228	3	47127752	G	A	D	Passenger	PASSENGER/OTHER		TRUE	TRUE	TRUE	Non-pathogenic	FALSE
MaPT03	*SF3B1*	p.Gly83Gly	synonymous_variant	8.80%	385		0.55	0.0006	0.3952		190	2	198285804	T	C	.	.	.		TRUE	TRUE	TRUE	Non-pathogenic	FALSE
MaPT04	*ANKRD11*	p.Thr1326Met	missense_variant	3.50%	227	LOH	0.06	0	0.024		170	16	89348973	G	A	N	Passenger	PASSENGER/OTHER					Non-pathogenic	FALSE
MaPT04	*ATRX*	p.Gln929Glu	missense_variant	4.50%	266		0.12	0	0.0638		151	X	76937963	G	C	P	Passenger	PASSENGER/OTHER		TRUE		TRUE	Non-pathogenic	FALSE
MaPT04	*KMT2D*	p.Trp2049*	stop_gained	39.00%	345		1	0.918	0.8698	Clonal	130	12	49435737	C	T	A	.	.		TRUE	TRUE	TRUE	Likely pathogenic	FALSE
MaPT04	*NF1*	.	splice_acceptor_variant&intron_variant	40.40%	267		1	0.9217	0.8685	Clonal	102	17	29508438	A	G	D	.	.		TRUE	TRUE	TRUE	Likely pathogenic	FALSE
MaPT04	*TSC2*	p.Leu191Leu	synonymous_variant	35.20%	259		0.93	0.7497	0.773	Clonal	98	16	2105494	C	T	.	.	.				TRUE	Non-pathogenic	FALSE
MaPT05	*ATM*	p.Asp1848del	inframe_deletion	35.67%	342		1	0.9429	0.9486	Clonal	278	11	108175444	CATG	C	D	.		Deleterious	TRUE	TRUE	TRUE	Likely pathogenic	FALSE
MaPT05	*EGFR*	p.Glu84Val	missense_variant	20.00%	220		1	0.83	0.7271	Clonal	233	7	55211008	A	T	D	Passenger	CANCER		TRUE	TRUE	TRUE	Likely pathogenic	FALSE
MaPT05	*PPP6C*	p.Arg301Leu	missense_variant	35.70%	572		1	0.9036	0.9677	Clonal	654	9	127912079	C	A	D	Passenger	PASSENGER/OTHER			TRUE		Non-pathogenic	FALSE
MaPT06	*ERBB2*	p.Val777Leu	missense_variant	3.40%	527		0.14	0	0.085		292	17	37881000	G	T	D	Driver	CANCER			TRUE	TRUE	Likely pathogenic	TRUE
MaPT06	*RB1*	p.Asp156fs	frameshift_variant	30.81%	244	LOH	0.97	0.8737	0.777	Clonal	211	13	48919301	GA	G	A	.			TRUE	TRUE	TRUE	Likely pathogenic	FALSE
MaPT06	*TP53*	p.Arg342*	stop_gained	40.60%	389	LOH	1	0.2076	0.9381	Clonal	276	17	7574003	G	A	A	.	.		TRUE	TRUE	TRUE	Likely pathogenic	FALSE
MaPT08	*NF1*	p.Gln2492*	stop_gained	79.80%	178	LOH	1	0	0.9646	Clonal	85	17	29679291	C	T	A	.	.		TRUE	TRUE	TRUE	Likely pathogenic	FALSE
MaPT08	*RB1*	p.Asn849Ile	missense_variant	55.10%	118	LOH	1	0.9384	0.8424	Clonal	73	13	49050862	A	T	D	Passenger	CANCER		TRUE	TRUE	TRUE	Likely pathogenic	FALSE
MaPT08	*TP53*	p.Arg342Pro	missense_variant	81.10%	254	LOH	1	0	0.975	Clonal	156	17	7574002	C	G	N	Driver	CANCER		TRUE	TRUE	TRUE	Likely pathogenic	FALSE
MaPT10	*NRAS*	p.Gln61Lys	missense_variant	41.00%	162		0.96	0.8275	0.7683	Clonal	197	1	115256530	G	T	D	Driver	CANCER		TRUE	TRUE	TRUE	Likely pathogenic	TRUE
MaPT10	*PIK3CA*	p.His1047Arg	missense_variant	37.60%	174		0.88	0.6117	0.7091	Clonal	183	3	178952085	A	G	D	Driver	CANCER		TRUE	TRUE	TRUE	Likely pathogenic	TRUE
MaPT10	*TERT*	c.-124C>T	upstream_gene_variant	51.90%	184		1	0.8633	0.9049	Clonal	135	5	1295228	G	A	.	.	.					Likely pathogenic	TRUE
MaPT10	*EGFR*	p.Leu62Arg	missense_variant	60.90%	332		1	0.9864	0.9736	Clonal	273	7	55210075	T	G	D	Driver	PASSENGER/OTHER		TRUE	TRUE	TRUE	Likely pathogenic	FALSE
MaPT10	*PDGFRB*	p.Arg561Cys	missense_variant	40.40%	208		0.95	0.8206	0.7833	Clonal	217	5	149505134	G	A	D	Passenger	CANCER				TRUE	Likely pathogenic	FALSE
MaPT10	*PIK3CA*	p.Glu453Gly	missense_variant	44.60%	139		1	0.902	0.8131	Clonal	135	3	178928080	A	G	D	Driver	CANCER		TRUE	TRUE	TRUE	Likely pathogenic	FALSE
MaPT10	*SETD2*	p.Gln1794fs	frameshift_variant	36.30%	286		0.85	0.3383	0.7227		208	3	47127702	G	GT	A	.			TRUE	TRUE	TRUE	Likely pathogenic	FALSE
MaPT12	*TERT*	c.-124C>T	upstream_gene_variant	19.40%	279		0.92	0.6904	0.7064	Clonal	176	5	1295228	G	A	.	.	.					Likely pathogenic	TRUE
MaPT12	*AMER1*	p.Asp537fs	frameshift_variant	11.01%	1122	LOH	0.42	0	0.348		722	X	63411556	GT	G	A	.					TRUE	Likely pathogenic	FALSE
MaPT12	*ATR*	p.Leu2091Ser	missense_variant	12.70%	300		0.48	0	0.3493		180	3	142188975	A	G	D	Driver	CANCER		TRUE			Likely pathogenic	FALSE
MaPT12	*TP53*	p.Tyr220*	stop_gained	17.80%	466		0.67	0.0002	0.5466		422	17	7578189	A	T	A	.	.		TRUE	TRUE	TRUE	Likely pathogenic	FALSE
MaPT19	*TERT*	c.-124C>T	upstream_gene_variant	46.38%	138		1	0.9679	0.9215	Clonal	107	5	1295228	G	A	.	.	.				TRUE	Likely pathogenic	TRUE
MaPT19	*EGFR*	p.Val774Met	missense_variant	40.39%	676		1	0.0174	0.9807	Clonal	259	7	55249022	G	A	D	Driver	CANCER		TRUE	TRUE	TRUE	Likely pathogenic	FALSE
MaPT19	*PTEN*	p.Tyr68His	missense_variant	41.48%	270	LOH	1	0.0039	0.9337	Clonal	218	10	89685307	T	C	D	Driver	CANCER		TRUE	TRUE	TRUE	Likely pathogenic	FALSE
MaPT19	*RUNX1*	p.Ser402Phe	missense_variant	16.67%	378		0.76	0.1033	0.5964		278	21	36164670	G	A	D	Passenger	.		TRUE	TRUE	TRUE	Non-pathogenic	FALSE
MaPT19	*RUNX1*	p.Tyr403fs	frameshift_variant	16.22%	376		0.74	0.0607	0.5781		276	21	36164653	CGTAGTACAGGTGGT	C	.	.			TRUE	TRUE	TRUE	Likely pathogenic	FALSE
MaPT20	*AKT1*	p.Glu17Lys	missense_variant	38.62%	378		1	0.8532	0.9208	Clonal	244	14	105246551	C	T	D	Driver	CANCER		TRUE	TRUE	TRUE	Likely pathogenic	TRUE
MaPT20	*TERT*	c.-124C>T	upstream_gene_variant	19.62%	158		0.61	0.0062	0.4341		192	5	1295228	G	A	.	.	.				TRUE	Likely pathogenic	TRUE
MaPT20	*TP53*	p.Arg249Ser	missense_variant	62.99%	489		1	0.9877	0.9815	Clonal	273	17	7577534	C	A	D	Driver	CANCER		TRUE	TRUE	TRUE	Likely pathogenic	TRUE
MaPT20	*KLF4*	p.Ser468Ser	synonymous_variant	32.47%	579		1	0.9248	0.8695	Clonal	345	9	110248068	C	T	D	.					TRUE	Non-pathogenic	FALSE
MaPT20	*MED12*	p.48_55del	inframe_deletion	25.27%	1021		0.78	0	0.6975		471	X	70339265	CAGCCTGCTGTCTCTGGGGATG	C	.	.		Deleterious		TRUE	TRUE	Likely pathogenic	FALSE
MaPT20	*MTOR*	p.Ser2013Gly	missense_variant	39.39%	325		1	0.8129	0.9166	Clonal	189	1	11187860	T	C	D	Passenger	CANCER		TRUE	TRUE		Likely pathogenic	FALSE
MaPT20	*NF1*	.	splice_acceptor_variant&intron_variant	73.85%	715		1	0.0001	0.9901	Clonal	299	17	29670025	A	C	D	.	.		TRUE	TRUE	TRUE	Likely pathogenic	FALSE
MaPT20	*NF2*	p.Glu527Val	missense_variant	59.68%	248	LOH	1	0.9533	0.9634	Clonal	164	22	30077433	A	T	D	Passenger	CANCER				TRUE	Likely pathogenic	FALSE
MaPT20	*RB1*	p.Leu607fs	frameshift_variant	54.11%	414	LOH	1	0.9257	0.9428	Clonal	266	13	49030344	CTT	C	A	.			TRUE	TRUE	TRUE	Likely pathogenic	FALSE
