# Supplementary material for: Phyllodes tumors with and without fibroadenoma-like areas display distinct genomic features and may evolve through distinct pathways
Source: NPJ Breast Cancer. 2017 Oct 12;3:40. doi: 10.1038/s41523-017-0042-6 (PMC5638820; doi:10.1038/s41523-017-0042-6)
Supplement: Supplementary file 5 — Supplementary Table 3 [file 41523_2017_42_MOESM5_ESM.pdf]

Supplementary Table 3: OncoKB level of evidence of clinical actionability of genetic alterations

Sample	Gene	Amino Acid Change	Effect	Hotspot	Breast Cancer	Ovarian Cancer	Thyroid Cancer	Melanoma	Colorectal Cancer	Endometrial Cancer	All Tumors
BoPT03	MED12	p.Glu172Lys	missense_variant		.	.	.	.	.	.	.
BoPT03	RARA	p.Cys235Trp	missense_variant		.	.	.	.	.	.	.
BoPT03	RB1	p.Gly310fs	frameshift_variant		.	.	.	.	.	.	.
BoPT03	RB1	p.Ile848Arg	missense_variant		.	.	.	.	.	.	.
BoPT03	RB1	p.Pro250fs	frameshift_variant		.	.	.	.	.	.	.
BoPT03	SETD2	p.Arg1625Cys	missense_variant		.	.	.	.	.	.	.
BoPT03	TERT	c.-124C>T	upstream_gene_variant	TRUE	.	.	.	.	.	.	.
BoPT05	DOT1L	p.Gly1471Glu	missense_variant		.	.	.	.	.	.	.
BoPT05	MED12	p.Leu39_Asn47del	inframe_deletion		.	.	.	.	.	.	.
BoPT05	TERT	c.-124C>T	upstream_gene_variant	TRUE	.	.	.	.	.	.	.
BoPT06	ASXL1	p.Glu1423Glu	synonymous_variant		.	.	.	.	.	.	.
BoPT06	EIF4A2	p.Met38Ile	missense_variant		.	.	.	.	.	.	.
BoPT06	IRS1	p.Ala874fs	frameshift_variant		.	.	.	.	.	.	.
BoPT06	MED12	p.Gly44Ala	missense_variant		.	.	.	.	.	.	.
BoPT06	RARA	p.Leu398Val	missense_variant		.	.	.	.	.	.	.
BoPT06	TRAF7	p.Asp357Glu	missense_variant		.	.	.	.	.	.	.
BoPT07	MED12	p.Gly44Val	missense_variant		.	.	.	.	.	.	.
BoPT07	RARA	p.Ser287Leu	missense_variant		.	.	.	.	.	.	.
BoPT07	TERT	c.-124C>T	upstream_gene_variant	TRUE	.	.	.	.	.	.	.
BoPT09	JAK3	p.Leu635His	missense_variant		.	.	.	.	.	.	.
BoPT09	KDR	p.Ala775Val	missense_variant		.	.	.	.	.	.	.
BoPT09	MED12	p.Gly44Val	missense_variant		.	.	.	.	.	.	.
BoPT09	PTPN11	p.Arg527His	missense_variant		.	.	.	.	.	.	.
MaPT01	EGFR	p.Gly63Arg	missense_variant		.	.	.	.	.	.	.
MaPT01	TERT	c.-124C>T	upstream_gene_variant	TRUE	.	.	.	.	.	.	.
MaPT10	EGFR	p.Leu62Arg	missense_variant		.	.	.	.	.	.	.
MaPT10	NRAS	p.Gln61Lys	missense_variant	TRUE	.	.	3A	3A	3A	.	.
MaPT10	PDGFRB	p.Arg561Cys	missense_variant		.	.	.	.	.	.	.
MaPT10	PIK3CA	p.Glu453Gly	missense_variant		.	.	.	.	.	.	.
MaPT10	PIK3CA	p.His1047Arg	missense_variant	TRUE	3A	4	.	.	.	4	4
MaPT10	SETD2	p.Gln1794fs	frameshift_variant		.	.	.	.	.	.	.
MaPT10	TERT	c.-124C>T	upstream_gene_variant	TRUE	.	.	.	.	.	.	.
MaPT12	AMER1	p.Asp537fs	frameshift_variant		.	.	.	.	.	.	.
MaPT12	ATR	p.Leu2091Ser	missense_variant		.	.	.	.	.	.	.
MaPT12	TERT	c.-124C>T	upstream_gene_variant	TRUE	.	.	.	.	.	.	.
MaPT12	TP53	p.Tyr220*	stop_gained		.	.	.	.	.	.	.
MaPT19	EGFR	p.Val774Met	missense_variant		.	.	.	.	.	.	.

MaPT19	PTEN	p.Tyr68His	missense_variant		.	.	.	.	.	.	.
MaPT19	RUNX1	p.Ser402Phe	missense_variant		.	.	.	.	.	.	.
MaPT19	RUNX1	p.Tyr403fs	frameshift_variant		.	.	.	.	.	.	.
MaPT19	TERT	c.-124C>T	upstream_gene_variant	TRUE	.	.	.	.	.	.	.
MaPT02	CDH1	p.Glu745*	stop_gained		.	.	.	.	.	.	.
MaPT02	ERBB3	p.Val104Leu	missense_variant	TRUE	.	.	.	.	.	.	.
MaPT02	FAT1	p.Ser377Ser	synonymous_variant		.	.	.	.	.	.	.
MaPT02	RB1	p.Cys706Phe	missense_variant		.	.	.	.	.	.	.
MaPT02	RET	p.Ser406Gly	missense_variant		.	.	.	.	.	.	.
MaPT02	RUNX1	p.Asp93Tyr	missense_variant		.	.	.	.	.	.	.
MaPT02	TP53	p.Ser241Tyr	missense_variant	TRUE	.	.	.	.	.	.	.
MaPT20	AKT1	p.Glu17Lys	missense_variant	TRUE	3A	3A	.	.	.	.	4
MaPT20	KLF4	p.Ser468Ser	synonymous_variant		.	.	.	.	.	.	.
MaPT20	MED12	p.48_55del	inframe_deletion		.	.	.	.	.	.	.
MaPT20	MTOR	p.Ser2013Gly	missense_variant		.	.	.	.	.	.	.
MaPT20	NF1	.	splice_acceptor_variant&intron_ variant		.	.	.	.	.	.	.
MaPT20	NF2	p.Glu527Val	missense_variant		.	.	.	.	.	.	.
MaPT20	RB1	p.Leu607fs	frameshift_variant		.	.	.	.	.	.	.
MaPT20	TERT	c.-124C>T	upstream_gene_variant	TRUE	.	.	.	.	.	.	.
MaPT20	TP53	p.Arg249Ser	missense_variant	TRUE	.	.	.	.	.	.	.
MaPT03	MED12	p.Gly44Asp	missense_variant		.	.	.	.	.	.	.
MaPT03	SETD2	p.Asp1616His	missense_variant		.	.	.	.	.	.	.
MaPT03	SETD2	p.Ser1777Phe	missense_variant		.	.	.	.	.	.	.
MaPT03	SF3B1	p.Gly83Gly	synonymous_variant		.	.	.	.	.	.	.
MaPT03	TERT	c.-124C>T	upstream_gene_variant	TRUE	.	.	.	.	.	.	.
MaPT04	ANKRD11	p.Thr1326Met	missense_variant		.	.	.	.	.	.	.
MaPT04	ATRX	p.Gln929Glu	missense_variant		.	.	.	.	.	.	.
MaPT04	KMT2D	p.Trp2049*	stop_gained		.	.	.	.	.	.	.
MaPT04	NF1	.	splice_acceptor_variant&intron_ variant		.	.	.	.	.	.	.
MaPT04	TSC2	p.Leu191Leu	synonymous_variant		.	.	.	.	.	.	.
MaPT05	ATM	p.Asp1848del	inframe_deletion		.	.	.	.	.	.	.
MaPT05	EGFR	p.Glu84Val	missense_variant		.	.	.	.	.	.	.
MaPT05	PPP6C	p.Arg301Leu	missense_variant		.	.	.	.	.	.	.
MaPT06	ERBB2	p.Val777Leu	missense_variant	TRUE	3A	.	.	.	.	.	.
MaPT06	RB1	p.Asp156fs	frameshift_variant		.	.	.	.	.	.	.
MaPT06	TP53	p.Arg342*	stop_gained		.	.	.	.	.	.	.
MaPT08	NF1	p.Gln2492*	stop_gained		.	.	.	.	.	.	.
MaPT08	RB1	p.Asn849Ile	missense_variant		.	.	.	.	.	.	.
MaPT08	TP53	p.Arg342Pro	missense_variant		.	.	.	.	.	.	.
