# Supplementary material for: Phyllodes tumors with and without fibroadenoma-like areas display distinct genomic features and may evolve through distinct pathways
Source: NPJ Breast Cancer. 2017 Oct 12;3:40. doi: 10.1038/s41523-017-0042-6 (PMC5638820; doi:10.1038/s41523-017-0042-6)
Supplement: Supplementary file 6 — Supplementary Table 4 [file 41523_2017_42_MOESM6_ESM.pdf]

Supplementary Table 4: List of genes included in the MSK-IMPACT panels employed in this study

HGNC symbol	Approved.Name	IMPACT410	IMPACTv3
ABL1	c-abl oncogene 1, non-receptor tyrosine kinase	TRUE	TRUE
ABL2	v-abl Abelson murine leukemia viral oncogene homolog 2		TRUE
ACVR1	activin A receptor, type I	TRUE	
AKT1	v-akt murine thymoma viral oncogene homolog 1	TRUE	TRUE
AKT2	v-akt murine thymoma viral oncogene homolog 2	TRUE	TRUE
AKT3	v-akt murine thymoma viral oncogene homolog 3 (protein kinase B, gamma)	TRUE	TRUE
ALK	anaplastic lymphoma receptor tyrosine kinase	TRUE	TRUE
ALOX12B	arachidonate 12-lipoxygenase, 12R type	TRUE	TRUE
AMER1	family with sequence similarity 123B	TRUE	TRUE
ANKRD11	ankyrin repeat domain 11	TRUE	
APC	adenomatous polyposis coli	TRUE	TRUE
AR	androgen receptor	TRUE	TRUE
ARAF	v-raf murine sarcoma 3611 viral oncogene homolog	TRUE	TRUE
ARHGAP26	Rho GTPase activating protein 26		TRUE
ARID1A	AT rich interactive domain 1A (SWI-like)	TRUE	TRUE
ARID1B	AT rich interactive domain 1B (SWI1-like)	TRUE	
ARID2	AT rich interactive domain 2 (ARID, RFX-like)	TRUE	TRUE
ARID5B	AT rich interactive domain 5B (MRF1-like)	TRUE	
ASXL1	additional sex combs like 1 (Drosophila)	TRUE	TRUE
ASXL2	additional sex combs like 2 (Drosophila)	TRUE	
ATM	ataxia telangiectasia mutated	TRUE	TRUE
ATR	ataxia telangiectasia and Rad3 related	TRUE	
ATRX	alpha thalassemia/mental retardation syndrome X-linked	TRUE	TRUE
AURKA	aurora kinase A	TRUE	TRUE
AURKB	aurora kinase B	TRUE	
AXIN1	axin 1	TRUE	
AXIN2	axin 2	TRUE	
AXL	AXL receptor tyrosine kinase	TRUE	
B2M	beta-2-microglobulin	TRUE	
BAP1	BRCA1 associated protein-1 (ubiquitin carboxy-terminal hydrolase)	TRUE	TRUE
BARD1	BRCA1 associated RING domain 1	TRUE	
BBC3	BCL2 binding component 3	TRUE	
BCL10	B-cell CLL/lymphoma 10	TRUE	
BCL2	B-cell CLL/lymphoma 2	TRUE	
BCL2L1	BCL2-like 1	TRUE	TRUE
BCL2L11	BCL2-like 11 (apoptosis facilitator)	TRUE	TRUE
BCL6	B-cell CLL/lymphoma 6	TRUE	TRUE
BCOR	BCL6 corepressor	TRUE	TRUE
BIRC2	baculoviral IAP repeat-containing 2		TRUE
BIRC3	baculoviral IAP repeat-containing 3	TRUE	

BLM	Bloom syndrome, RecQ helicase-like	TRUE	
BMPR1A	bone morphogenetic protein receptor, type IA	TRUE	
BRAF	v-raf murine sarcoma viral oncogene homolog B1	TRUE	TRUE
BRCA1	breast cancer 1, early onset	TRUE	TRUE
BRCA2	breast cancer 2, early onset	TRUE	TRUE
BRD4	bromodomain containing 4	TRUE	
BRIP1	BRCA1 interacting protein C-terminal helicase 1	TRUE	
BTK	Bruton agammaglobulinemia tyrosine kinase	TRUE	
BUB1B	budding uninhibited by benzimidazoles 1 homolog beta (yeast)		TRUE
CALR	calreticulin	TRUE	
CARD11	caspase recruitment domain family, member 11	TRUE	TRUE
CASP8	caspase 8, apoptosis-related cysteine peptidase	TRUE	
CBFB	core-binding factor, beta subunit	TRUE	
CBL	Cas-Br-M (murine) ecotropic retroviral transforming sequence	TRUE	TRUE
CBLB	Cas-Br-M (murine) ecotropic retroviral transforming sequence b		TRUE
CCND1	cyclin D1	TRUE	TRUE
CCND2	cyclin D2	TRUE	
CCND3	cyclin D3	TRUE	
CCNE1	cyclin E1	TRUE	TRUE
CD274	CD274 molecule	TRUE	
CD276	CD276 molecule	TRUE	
CD79A	CD79a molecule, immunoglobulin-associated alpha	TRUE	
CD79B	CD79b molecule, immunoglobulin-associated beta	TRUE	TRUE
CDC42EP2	CDC42 effector protein (Rho GTPase binding) 2		TRUE
CDC73	cell division cycle 73, Paf1/RNA polymerase II complex component, homolog (S. cerevisiae)	TRUE	TRUE
CDH1	cadherin 1, type 1, E-cadherin (epithelial)	TRUE	TRUE
CDH11	cadherin 11, type 2, OB-cadherin (osteoblast)		TRUE
CDK12	cyclin-dependent kinase 12	TRUE	TRUE
CDK4	cyclin-dependent kinase 4	TRUE	TRUE
CDK6	cyclin-dependent kinase 6	TRUE	TRUE
CDK8	cyclin-dependent kinase 8	TRUE	TRUE
CDKN1A	cyclin-dependent kinase inhibitor 1A (p21, Cip1)	TRUE	TRUE
CDKN1B	cyclin-dependent kinase inhibitor 1B (p27, Kip1)	TRUE	
CDKN2A	cyclin-dependent kinase inhibitor 2A (melanoma, p16, inhibits CDK4)	TRUE	TRUE
CDKN2B	cyclin-dependent kinase inhibitor 2B (p15, inhibits CDK4)	TRUE	TRUE
CDKN2C	cyclin-dependent kinase inhibitor 2C (p18, inhibits CDK4)	TRUE	TRUE
CEBPA	CCAAT/enhancer binding protein (C/EBP), alpha	TRUE	TRUE
CENPA	centromere protein A	TRUE	
CHEK1	CHK1 checkpoint homolog (S. pombe)	TRUE	TRUE
CHEK2	CHK2 checkpoint homolog (S. pombe)	TRUE	TRUE
CIC	capicua homolog (Drosophila)	TRUE	TRUE
CREBBP	CREB binding protein	TRUE	TRUE
CRKL	v-crk sarcoma virus CT10 oncogene homolog (avian)-like	TRUE	TRUE
CRLF2	cytokine receptor-like factor 2	TRUE	TRUE

CSF1R	colony stimulating factor 1 receptor	TRUE	TRUE
CSF3R	colony stimulating factor 3 receptor (granulocyte)	TRUE	
CTCF	CCCTC-binding factor (zinc finger protein)	TRUE	
CTLA4	cytotoxic T-lymphocyte-associated protein 4	TRUE	
CTNNB1	catenin (cadherin-associated protein), beta 1, 88kDa	TRUE	TRUE
CUL3	cullin 3	TRUE	
CXCR4	chemokine (C-X-C motif) receptor 4	TRUE	
CYLD	cylindromatosis (turban tumor syndrome)		TRUE
DAXX	death-domain associated protein	TRUE	TRUE
DCUN1D1	DCN1, defective in cullin neddylation 1, domain containing 1 (S. cerevisiae)	TRUE	
DDR2	discoidin domain receptor tyrosine kinase 2	TRUE	TRUE
DICER1	dicer 1, ribonuclease type III	TRUE	TRUE
DIS3	DIS3 mitotic control homolog (S. cerevisiae)	TRUE	TRUE
DNAJB1	DnaJ (Hsp40) homolog, subfamily B, member 1	TRUE	
DNMT1	DNA (cytosine-5-)-methyltransferase 1	TRUE	TRUE
DNMT3A	DNA (cytosine-5-)-methyltransferase 3 alpha	TRUE	TRUE
DNMT3B	DNA (cytosine-5-)-methyltransferase 3 beta	TRUE	TRUE
DOT1L	DOT1-like, histone H3 methyltransferase (S. cerevisiae)	TRUE	
E2F3	E2F transcription factor 3	TRUE	TRUE
EED	embryonic ectoderm development	TRUE	
EGFL7	EGF-like-domain, multiple 7	TRUE	
EGFR	epidermal growth factor receptor	TRUE	TRUE
EIF1AX	eukaryotic translation initiation factor 1A, X-linked	TRUE	
EIF4A2	eukaryotic translation initiation factor 4A2	TRUE	
EIF4E	eukaryotic translation initiation factor 4E	TRUE	
EIF4EBP1	eukaryotic translation initiation factor 4E binding protein 1		TRUE
EP300	E1A binding protein p300	TRUE	TRUE
EPCAM	epithelial cell adhesion molecule	TRUE	
EPHA10	EPH receptor A10		TRUE
EPHA2	EPH receptor A2		TRUE
EPHA3	EPH receptor A3	TRUE	TRUE
EPHA4	EPH receptor A4		TRUE
EPHA5	EPH receptor A5	TRUE	TRUE
EPHA6	EPH receptor A6		TRUE
EPHA7	EPH receptor A7	TRUE	TRUE
EPHA8	EPH receptor A8		TRUE
EPHB1	EPH receptor B1	TRUE	TRUE
EPHB2	EPH receptor B2		TRUE
EPHB3	EPH receptor B3		TRUE
EPHB4	EPH receptor B4		TRUE
EPHB6	EPH receptor B6		TRUE
ERBB2	v-erb-b2 erythroblastic leukemia viral oncogene homolog 2, neuro/glioblastoma derived oncogene homolog (avian)	TRUE	TRUE
ERBB3	v-erb-b2 erythroblastic leukemia viral oncogene homolog 3 (avian)	TRUE	TRUE
ERBB4	v-erb-a erythroblastic leukemia viral oncogene homolog 4 (avian)	TRUE	TRUE

ERCC2	excision repair cross-complementing rodent repair deficiency, complementation group 2	TRUE	
ERCC3	excision repair cross-complementing rodent repair deficiency, complementation group 3 (xeroderma pigmentosum group B complementing)	TRUE	
ERCC4	excision repair cross-complementing rodent repair deficiency, complementation group 4	TRUE	
ERCC5	excision repair cross-complementing rodent repair deficiency, complementation group 5	TRUE	
ERG	v-ets erythroblastosis virus E26 oncogene homolog (avian)	TRUE	TRUE
ERRFI1	ERBB receptor feedback inhibitor 1	TRUE	
ESR1	estrogen receptor 1	TRUE	TRUE
ETV1	ets variant 1	TRUE	TRUE
ETV6	ets variant 6	TRUE	TRUE
EZH2	enhancer of zeste homolog 2 (Drosophila)	TRUE	TRUE
FAM175A	family with sequence similarity 175, member A	TRUE	
FAM46C	family with sequence similarity 46, member C	TRUE	TRUE
FANCA	Fanconi anemia, complementation group A	TRUE	
FANCC	Fanconi anemia, complementation group C	TRUE	
FAS	Fas (TNF receptor superfamily, member 6)		TRUE
FAT1	FAT tumor suppressor homolog 1 (Drosophila)	TRUE	TRUE
FBXO11	F-box protein 11		TRUE
FBXW7	F-box and WD repeat domain containing 7	TRUE	TRUE
FGF19	fibroblast growth factor 19	TRUE	
FGF3	fibroblast growth factor 3	TRUE	
FGF4	fibroblast growth factor 4	TRUE	
FGFR1	fibroblast growth factor receptor 1	TRUE	TRUE
FGFR2	fibroblast growth factor receptor 2	TRUE	TRUE
FGFR3	fibroblast growth factor receptor 3	TRUE	TRUE
FGFR4	fibroblast growth factor receptor 4	TRUE	TRUE
FH	fumarate hydratase	TRUE	TRUE
FLCN	folliculin	TRUE	TRUE
FLT1	fms-related tyrosine kinase 1 (vascular endothelial growth factor/vascular permeability factor receptor)	TRUE	TRUE
FLT3	fms-related tyrosine kinase 3	TRUE	TRUE
FLT4	fms-related tyrosine kinase 4	TRUE	TRUE
FOXA1	forkhead box A1	TRUE	
FOXL2	forkhead box L2	TRUE	TRUE
FOXO1	forkhead box O1	TRUE	
FOXP1	forkhead box P1	TRUE	
FUBP1	far upstream element (FUSE) binding protein 1	TRUE	TRUE
FYN	FYN oncogene related to SRC, FGR, YES	TRUE	
GATA1	GATA binding protein 1 (globin transcription factor 1)	TRUE	TRUE
GATA2	GATA binding protein 2	TRUE	TRUE
GATA3	GATA binding protein 3	TRUE	TRUE
GLI1	GLI family zinc finger 1	TRUE	TRUE
GLI3	GLI family zinc finger 3		TRUE
GNA11	guanine nucleotide binding protein (G protein), alpha 11 (Gq class)	TRUE	TRUE
GNAQ	guanine nucleotide binding protein (G protein), q polypeptide	TRUE	TRUE
GNAS	GNAS complex locus	TRUE	TRUE

GOLPH3	golgi phosphoprotein 3 (coat-protein)		TRUE
GPS2	G protein pathway suppressor 2	TRUE	
GREM1	gremlin 1	TRUE	
GRIN2A	glutamate receptor, ionotropic, N-methyl D-aspartate 2A	TRUE	TRUE
GRM3	glutamate receptor, metabotropic 3		TRUE
GSK3B	glycogen synthase kinase 3 beta	TRUE	TRUE
H3F3A	H3 histone, family 3A	TRUE	
H3F3B	H3 histone, family 3B (H3.3B)	TRUE	
H3F3C	H3 histone, family 3C	TRUE	
HDAC2	histone deacetylase 2		TRUE
HGF	hepatocyte growth factor (hepapoietin A; scatter factor)	TRUE	
HIF1A	hypoxia inducible factor 1, alpha subunit (basic helix-loop-helix transcription factor)		TRUE
HIST1H1C	histone cluster 1, H1c	TRUE	
HIST1H2BD	histone cluster 1, H2bd	TRUE	
HIST1H3A	histone cluster 1, H3a	TRUE	
HIST1H3B	histone cluster 1, H3b	TRUE	
HIST1H3C	histone cluster 1, H3c	TRUE	
HIST1H3D	histone cluster 1, H3d	TRUE	
HIST1H3E	histone cluster 1, H3e	TRUE	
HIST1H3F	histone cluster 1, H3f	TRUE	
HIST1H3G	histone cluster 1, H3g	TRUE	
HIST1H3H	histone cluster 1, H3h	TRUE	
HIST1H3I	histone cluster 1, H3i	TRUE	
HIST1H3J	histone cluster 1, H3j	TRUE	
HIST2H3C	histone cluster 2, H3c	TRUE	
HIST2H3D	histone cluster 2, H3d	TRUE	
HIST3H3	histone cluster 3, H3	TRUE	
HLA-A	major histocompatibility complex, class I, A	TRUE	
HMGA2	high mobility group AT-hook 2		TRUE
HNF1A	HNF1 homeobox A	TRUE	TRUE
HOXB13	homeobox B13	TRUE	
HRAS	v-Ha-ras Harvey rat sarcoma viral oncogene homolog	TRUE	TRUE
HSP90AA1	heat shock protein 90kDa alpha (cytosolic), class A member 1		TRUE
ICOSLG	inducible T-cell co-stimulator ligand	TRUE	
ID3	inhibitor of DNA binding 3, dominant negative helix-loop-helix protein	TRUE	
IDH1	isocitrate dehydrogenase 1 (NADP+), soluble	TRUE	TRUE
IDH2	isocitrate dehydrogenase 2 (NADP+), mitochondrial	TRUE	TRUE
IFNGR1	interferon gamma receptor 1	TRUE	
IGF1	insulin-like growth factor 1 (somatomedin C)	TRUE	
IGF1R	insulin-like growth factor 1 receptor	TRUE	TRUE
IGF2	insulin-like growth factor 2 (somatomedin A)	TRUE	
IGFBP7	insulin-like growth factor binding protein 7		TRUE
IKBKE	inhibitor of kappa light polypeptide gene enhancer in B-cells, kinase epsilon	TRUE	TRUE
IKZF1	IKAROS family zinc finger 1 (Ikaros)	TRUE	TRUE

IL10	interleukin 10	TRUE	
IL7R	interleukin 7 receptor	TRUE	TRUE
INHA	inhibin, alpha	TRUE	
INHBA	inhibin, beta A	TRUE	
INPP4A	inositol polyphosphate-4-phosphatase, type I, 107kDa	TRUE	TRUE
INPP4B	inositol polyphosphate-4-phosphatase, type II, 105kDa	TRUE	TRUE
INSR	insulin receptor	TRUE	TRUE
IRF4	interferon regulatory factor 4	TRUE	
IRS1	insulin receptor substrate 1	TRUE	TRUE
IRS2	insulin receptor substrate 2	TRUE	TRUE
JAK1	Janus kinase 1	TRUE	TRUE
JAK2	Janus kinase 2	TRUE	TRUE
JAK3	Janus kinase 3	TRUE	TRUE
JUN	jun proto-oncogene	TRUE	TRUE
KCNJ5	potassium inwardly-rectifying channel, subfamily J, member 5		TRUE
KDM5A	lysine (K)-specific demethylase 5A	TRUE	
KDM5C	lysine (K)-specific demethylase 5C	TRUE	TRUE
KDM6A	lysine (K)-specific demethylase 6A	TRUE	TRUE
KDR	kinase insert domain receptor (a type III receptor tyrosine kinase)	TRUE	TRUE
KEAP1	kelch-like ECH-associated protein 1	TRUE	TRUE
KIT	v-kit Hardy-Zuckerman 4 feline sarcoma viral oncogene homolog	TRUE	TRUE
KLF4	Kruppel-like factor 4 (gut)	TRUE	
KLF6	Kruppel-like factor 6		TRUE
KMT2A	myeloid/lymphoid or mixed-lineage leukemia (trithorax homolog, Drosophila)	TRUE	TRUE
KMT2C	myeloid/lymphoid or mixed-lineage leukemia 3	TRUE	TRUE
KMT2D	myeloid/lymphoid or mixed-lineage leukemia 2	TRUE	TRUE
KRAS	v-Ki-ras2 Kirsten rat sarcoma viral oncogene homolog	TRUE	TRUE
LATS1	LATS, large tumor suppressor, homolog 1 (Drosophila)	TRUE	
LATS2	LATS, large tumor suppressor, homolog 2 (Drosophila)	TRUE	
LDHA	lactate dehydrogenase A		TRUE
LGR6	leucine-rich repeat-containing G protein-coupled receptor 6		TRUE
LMO1	LIM domain only 1 (rhombotin 1)	TRUE	TRUE
MAGI2	membrane associated guanylate kinase, WW and PDZ domain containing 2		TRUE
MALT1	mucosa associated lymphoid tissue lymphoma translocation gene 1	TRUE	
MAP2K1	mitogen-activated protein kinase kinase 1	TRUE	TRUE
MAP2K2	mitogen-activated protein kinase kinase 2	TRUE	TRUE
MAP2K4	mitogen-activated protein kinase kinase 4	TRUE	TRUE
MAP3K1	mitogen-activated protein kinase kinase kinase 1	TRUE	
MAP3K13	mitogen-activated protein kinase kinase kinase 13	TRUE	
MAP3K14	mitogen-activated protein kinase kinase kinase 14	TRUE	
MAP3K8	mitogen-activated protein kinase kinase kinase 8		TRUE
MAPK1	mitogen-activated protein kinase 1	TRUE	
MAPK3	mitogen-activated protein kinase 3	TRUE	
MAX	MYC associated factor X	TRUE	

MCL1	myeloid cell leukemia sequence 1 (BCL2-related)	TRUE	TRUE
MDC1	mediator of DNA-damage checkpoint 1	TRUE	
MDM2	Mdm2 p53 binding protein homolog (mouse)	TRUE	TRUE
MDM4	Mdm4 p53 binding protein homolog (mouse)	TRUE	TRUE
MED12	mediator complex subunit 12	TRUE	TRUE
MEF2B	myocyte enhancer factor 2B	TRUE	TRUE
MEN1	multiple endocrine neoplasia I	TRUE	TRUE
MET	met proto-oncogene (hepatocyte growth factor receptor)	TRUE	TRUE
MGA	MAX gene associated	TRUE	
MITF	microphthalmia-associated transcription factor	TRUE	TRUE
MLH1	mutL homolog 1, colon cancer, nonpolyposis type 2 (E. coli)	TRUE	TRUE
MLST8	MTOR associated protein, LST8 homolog (S. cerevisiae)		TRUE
MPL	myeloproliferative leukemia virus oncogene	TRUE	TRUE
MRE11A	MRE11 meiotic recombination 11 homolog A (S. cerevisiae)	TRUE	
MSH2	mutS homolog 2, colon cancer, nonpolyposis type 1 (E. coli)	TRUE	TRUE
MSH6	mutS homolog 6 (E. coli)	TRUE	TRUE
MST1	macrophage stimulating 1 (hepatocyte growth factor-like)	TRUE	
MST1R	macrophage stimulating 1 receptor (c-met-related tyrosine kinase)	TRUE	
MTOR	mechanistic target of rapamycin (serine/threonine kinase)	TRUE	TRUE
MUTYH	mutY homolog (E. coli)	TRUE	
MYB	v-myb myeloblastosis viral oncogene homolog (avian)		TRUE
MYC	v-myc myelocytomatosis viral oncogene homolog (avian)	TRUE	TRUE
MYCL	v-myc myelocytomatosis viral oncogene homolog 1, lung carcinoma derived (avian)	TRUE	TRUE
MYCN	v-myc myelocytomatosis viral related oncogene, neuroblastoma derived (avian)	TRUE	TRUE
MYD88	myeloid differentiation primary response gene (88)	TRUE	TRUE
MYOD1	myogenic differentiation 1	TRUE	
NBN	nibrin	TRUE	
NCOA2	nuclear receptor coactivator 2		TRUE
NCOA3	nuclear receptor coactivator 3	TRUE	
NCOR1	nuclear receptor corepressor 1	TRUE	
NEGR1	neuronal growth regulator 1	TRUE	
NF1	neurofibromin 1	TRUE	TRUE
NF2	neurofibromin 2 (merlin)	TRUE	TRUE
NFE2L2	nuclear factor (erythroid-derived 2)-like 2	TRUE	TRUE
NFKB1	nuclear factor of kappa light polypeptide gene enhancer in B-cells 1		TRUE
NFKB2	nuclear factor of kappa light polypeptide gene enhancer in B-cells 2 (p49/p100)		TRUE
NFKBIA	nuclear factor of kappa light polypeptide gene enhancer in B-cells inhibitor, alpha	TRUE	
NKX2-1	NK2 homeobox 1	TRUE	TRUE
NKX3-1	NK3 homeobox 1	TRUE	
NOTCH1	notch 1	TRUE	TRUE
NOTCH2	notch 2	TRUE	TRUE
NOTCH3	notch 3	TRUE	TRUE
NOTCH4	notch 4	TRUE	TRUE
NPM1	nucleophosmin (nucleolar phosphoprotein B23, numatrin)	TRUE	TRUE

NRAS	neuroblastoma RAS viral (v-ras) oncogene homolog	TRUE	TRUE
NSD1	nuclear receptor binding SET domain protein 1	TRUE	
NTRK1	neurotrophic tyrosine kinase, receptor, type 1	TRUE	TRUE
NTRK2	neurotrophic tyrosine kinase, receptor, type 2	TRUE	TRUE
NTRK3	neurotrophic tyrosine kinase, receptor, type 3	TRUE	TRUE
NUP93	nucleoporin 93kDa	TRUE	
PAK1	p21 protein (Cdc42/Rac)-activated kinase 1	TRUE	
PAK7	p21 protein (Cdc42/Rac)-activated kinase 7	TRUE	TRUE
PALB2	partner and localizer of BRCA2	TRUE	TRUE
PARK2	parkinson protein 2, E3 ubiquitin protein ligase (parkin)	TRUE	TRUE
PARP1	poly (ADP-ribose) polymerase 1	TRUE	TRUE
PAX5	paired box 5	TRUE	TRUE
PBRM1	polybromo 1	TRUE	TRUE
PDCD1	programmed cell death 1	TRUE	
PDGFRA	platelet-derived growth factor receptor, alpha polypeptide	TRUE	TRUE
PDGFRB	platelet-derived growth factor receptor, beta polypeptide	TRUE	TRUE
PDPK1	3-phosphoinositide dependent protein kinase-1	TRUE	
PGR	progesterone receptor	TRUE	
PHOX2B	paired-like homeobox 2b	TRUE	TRUE
PIK3C2G	phosphoinositide-3-kinase, class 2, gamma polypeptide	TRUE	TRUE
PIK3C3	phosphoinositide-3-kinase, class 3	TRUE	
PIK3CA	phosphoinositide-3-kinase, catalytic, alpha polypeptide	TRUE	TRUE
PIK3CB	phosphoinositide-3-kinase, catalytic, beta polypeptide	TRUE	TRUE
PIK3CD	phosphoinositide-3-kinase, catalytic, delta polypeptide	TRUE	TRUE
PIK3CG	phosphoinositide-3-kinase, catalytic, gamma polypeptide	TRUE	TRUE
PIK3R1	phosphoinositide-3-kinase, regulatory subunit 1 (alpha)	TRUE	TRUE
PIK3R2	phosphoinositide-3-kinase, regulatory subunit 2 (beta)	TRUE	TRUE
PIK3R3	phosphoinositide-3-kinase, regulatory subunit 3 (gamma)	TRUE	TRUE
PIM1	pim-1 oncogene	TRUE	
PKM	pyruvate kinase, muscle		TRUE
PLCG2	phospholipase C, gamma 2 (phosphatidylinositol-specific)	TRUE	
PLK2	polo-like kinase 2	TRUE	TRUE
PMAIP1	phorbol-12-myristate-13-acetate-induced protein 1	TRUE	
PMS1	PMS1 postmeiotic segregation increased 1 (S. cerevisiae)	TRUE	
PMS2	PMS2 postmeiotic segregation increased 2 (S. cerevisiae)	TRUE	
PNRC1	proline-rich nuclear receptor coactivator 1	TRUE	TRUE
POLD1	polymerase (DNA directed), delta 1, catalytic subunit 125kDa	TRUE	
POLE	polymerase (DNA directed), epsilon	TRUE	
PPM1D	protein phosphatase, Mg2+/Mn2+ dependent, 1D	TRUE	
PPP2R1A	protein phosphatase 2, regulatory subunit A, alpha	TRUE	TRUE
PPP6C	protein phosphatase 6, catalytic subunit	TRUE	
PRDM1	PR domain containing 1, with ZNF domain	TRUE	TRUE
PREX2	phosphatidylinositol-3,4,5-trisphosphate-dependent Rac exchange factor 2		TRUE
PRKAA2	protein kinase, AMP-activated, alpha 2 catalytic subunit		TRUE

PRKAR1A	protein kinase, cAMP-dependent, regulatory, type I, alpha (tissue specific extinguisher 1)	TRUE	TRUE
PRKCI	protein kinase C, iota		TRUE
PTCH1	patched 1	TRUE	TRUE
PTEN	phosphatase and tensin homolog	TRUE	TRUE
PTPN11	protein tyrosine phosphatase, non-receptor type 11	TRUE	TRUE
PTPRD	protein tyrosine phosphatase, receptor type, D	TRUE	TRUE
PTPRS	protein tyrosine phosphatase, receptor type, S	TRUE	TRUE
PTPRT	protein tyrosine phosphatase, receptor type, T	TRUE	TRUE
RAB35	RAB35, member RAS oncogene family	TRUE	
RAC1	ras-related C3 botulinum toxin substrate 1 (rho family, small GTP binding protein Rac1)	TRUE	
RAD21	RAD21 homolog (S. pombe)	TRUE	
RAD50	RAD50 homolog (S. cerevisiae)	TRUE	
RAD51	RAD51 homolog (RecA homolog, E. coli) (S. cerevisiae)	TRUE	
RAD51B	RAD51-like 1 (S. cerevisiae)	TRUE	
RAD51C	RAD51 homolog C (S. cerevisiae)	TRUE	
RAD51D	RAD51-like 3 (S. cerevisiae)	TRUE	
RAD52	RAD52 homolog (S. cerevisiae)	TRUE	
RAD54L	RAD54-like (S. cerevisiae)	TRUE	
RAF1	v-raf-1 murine leukemia viral oncogene homolog 1	TRUE	TRUE
RARA	retinoic acid receptor, alpha	TRUE	TRUE
RASA1	RAS p21 protein activator (GTPase activating protein) 1	TRUE	
RB1	retinoblastoma 1	TRUE	TRUE
RBM10	RNA binding motif protein 10	TRUE	
RECQL4	RecQ protein-like 4	TRUE	
REL	v-rel reticuloendotheliosis viral oncogene homolog (avian)	TRUE	TRUE
RET	ret proto-oncogene	TRUE	TRUE
RFWD2	ring finger and WD repeat domain 2	TRUE	
RHEB	Ras homolog enriched in brain	TRUE	
RHOA	ras homolog gene family, member A	TRUE	
RICTOR	RPTOR independent companion of MTOR, complex 2	TRUE	TRUE
RIT1	Ras-like without CAAX 1	TRUE	
RNF43	ring finger protein 43	TRUE	TRUE
ROR2	receptor tyrosine kinase-like orphan receptor 2		TRUE
ROS1	c-ros oncogene 1 , receptor tyrosine kinase	TRUE	TRUE
RPS6KA4	ribosomal protein S6 kinase, 90kDa, polypeptide 4	TRUE	
RPS6KB2	ribosomal protein S6 kinase, 70kDa, polypeptide 2	TRUE	
RPTOR	regulatory associated protein of MTOR, complex 1	TRUE	TRUE
RUNX1	runt-related transcription factor 1	TRUE	TRUE
RYBP	RING1 and YY1 binding protein	TRUE	
SDHA	succinate dehydrogenase complex, subunit A, flavoprotein (Fp)	TRUE	
SDHAF2	succinate dehydrogenase complex assembly factor 2	TRUE	
SDHB	succinate dehydrogenase complex, subunit B, iron sulfur (Ip)	TRUE	TRUE
SDHC	succinate dehydrogenase complex, subunit C, integral membrane protein, 15kDa	TRUE	
SDHD	succinate dehydrogenase complex, subunit D, integral membrane protein	TRUE	

SETD2	SET domain containing 2	TRUE	TRUE
SF3B1	splicing factor 3b, subunit 1, 155kDa	TRUE	TRUE
SH2B3	SH2B adaptor protein 3	TRUE	
SH2D1A	SH2 domain containing 1A	TRUE	
SHQ1	SHQ1 homolog (S. cerevisiae)	TRUE	TRUE
SMAD2	SMAD family member 2	TRUE	
SMAD3	SMAD family member 3	TRUE	
SMAD4	SMAD family member 4	TRUE	TRUE
SMARCA4	SWI/SNF related, matrix associated, actin dependent regulator of chromatin, subfamily a, member 4	TRUE	TRUE
SMARCB1	SWI/SNF related, matrix associated, actin dependent regulator of chromatin, subfamily b, member 1	TRUE	TRUE
SMARCD1	SWI/SNF related, matrix associated, actin dependent regulator of chromatin, subfamily d, member 1	TRUE	
SMO	smoothened homolog (Drosophila)	TRUE	TRUE
SOCS1	suppressor of cytokine signaling 1	TRUE	TRUE
SOX17	SRY (sex determining region Y)-box 17	TRUE	
SOX2	SRY (sex determining region Y)-box 2	TRUE	TRUE
SOX9	SRY (sex determining region Y)-box 9	TRUE	
SPEN	spen homolog, transcriptional regulator (Drosophila)	TRUE	
SPOP	speckle-type POZ protein	TRUE	TRUE
SRC	v-src sarcoma (Schmidt-Ruppin A-2) viral oncogene homolog (avian)	TRUE	TRUE
SRSF2	serine/arginine-rich splicing factor 2	TRUE	TRUE
STAG2	stromal antigen 2	TRUE	TRUE
STAT3	signal transducer and activator of transcription 3 (acute-phase response factor)	TRUE	
STAT5A	signal transducer and activator of transcription 5A	TRUE	
STAT5B	signal transducer and activator of transcription 5B	TRUE	
STK11	serine/threonine kinase 11	TRUE	TRUE
STK40	serine/threonine kinase 40	TRUE	
SUFU	suppressor of fused homolog (Drosophila)	TRUE	TRUE
SUZ12	suppressor of zeste 12 homolog (Drosophila)	TRUE	
SYK	spleen tyrosine kinase	TRUE	
TBK1	TANK-binding kinase 1		TRUE
TBX3	T-box 3	TRUE	
TCEB1	transcription elongation factor B (SIII), polypeptide 1 (15kDa, elongin C)	TRUE	
TCF3	transcription factor 3 (E2A immunoglobulin enhancer binding factors E12/E47)	TRUE	
TCF7L2	transcription factor 7-like 2 (T-cell specific, HMG-box)	TRUE	
TEK	TEK tyrosine kinase, endothelial		TRUE
TERT	telomerase reverse transcriptase	TRUE	TRUE
TET1	tet oncogene 1	TRUE	TRUE
TET2	tet oncogene family member 2	TRUE	TRUE
TGFBR1	transforming growth factor, beta receptor 1	TRUE	
TGFBR2	transforming growth factor, beta receptor II (70/80kDa)	TRUE	TRUE
TMEM127	transmembrane protein 127	TRUE	
TMPRSS2	transmembrane protease, serine 2	TRUE	TRUE
TNFAIP3	tumor necrosis factor, alpha-induced protein 3	TRUE	TRUE
TNFRSF14	tumor necrosis factor receptor superfamily, member 14 (herpesvirus entry mediator)	TRUE	TRUE

TOP1	topoisomerase (DNA) I	TRUE	TRUE
TP53	tumor protein p53	TRUE	TRUE
TP63	tumor protein p63	TRUE	TRUE
TRAF2	TNF receptor-associated factor 2	TRUE	
TRAF7	TNF receptor-associated factor 7	TRUE	
TSC1	tuberous sclerosis 1	TRUE	TRUE
TSC2	tuberous sclerosis 2	TRUE	TRUE
TSHR	thyroid stimulating hormone receptor	TRUE	TRUE
U2AF1	U2 small nuclear RNA auxiliary factor 1	TRUE	TRUE
VEGFA	vascular endothelial growth factor A	TRUE	
VHL	von Hippel-Lindau tumor suppressor	TRUE	TRUE
VTCN1	V-set domain containing T cell activation inhibitor 1	TRUE	
WAS	Wiskott-Aldrich syndrome (eczema-thrombocytopenia)		TRUE
WNK1	WNK lysine deficient protein kinase 1		TRUE
WT1	Wilms tumor 1	TRUE	TRUE
XIAP	X-linked inhibitor of apoptosis	TRUE	
XPO1	exportin 1 (CRM1 homolog, yeast)	TRUE	TRUE
XRCC2	X-ray repair complementing defective repair in Chinese hamster cells 2	TRUE	
YAP1	Yes-associated protein 1	TRUE	TRUE
YES1	v-yes-1 Yamaguchi sarcoma viral oncogene homolog 1	TRUE	TRUE
ZFHX3	zinc finger homeobox 3	TRUE	
ZRSR2	zinc finger (CCCH type), RNA-binding motif and serine/arginine rich 2	TRUE	TRUE
